# The evolution law of deviatoric stress and asymmetric control technology in roadways during panel mining through overlying residual coal pillars

**DOI:** 10.1038/s41598-024-55242-y

**Published:** 2024-02-23

**Authors:** Chunyang Tian, Qiucheng Ye, Bohao Qi, Wenke Guo, Bowen Li, Xiangxiang Yan

**Affiliations:** 1 School of Energy and Mining Engineering, China University of Mining and Technology-Beijing, Beijing, 100083 China; 2 Beijing Tiandi Huatai Mining Management Co., Ltd, Beijing, China

**Keywords:** Residual coal pillar, Deviatoric stress, Partition support, Asymmetric support, Close-distance coal seam, Energy science and technology, Engineering

## Abstract

Close-distance coal seams (CDCS) are widely distributed, and the layout of the upper and lower panels can be divided into “=” type and “+” type. The “+” superposition of upper and lower coal pillars in CDCS caused strong mine pressure, but there are few studies on the panel crossing residual coal pillars (RCP) when the upper and lower coal seams are “+” type layout. In view of the special spatial position (“+” type layout), this paper takes the typical panel 4-301 of a particular mine as the project indagation background and studies mining and crossing the overlying coal pillars by dint of field measurement, numerical simulation, indoor test, and engineering application. Compared with vertical stress or horizontal stress alone, the indexes of deviatoric stress and plastic zone can reflect the failure evolution of surrounding rock more comprehensively. Hence, this paper analyzes the expansion form of the plastic zone and the variation law of deviatoric stress before and after mining influence in the underlying mining roadway. The research results show that: (1) There is a sub-peak zone of deviatoric stress under the RCP. The deviatoric stress is bimodal in the range of 9 m below. After the peak value decays to 7.4 MPa, it changes to a single peak located in the area directly below the middle of the RCP. (2) The maximum plastic zones of the roof and two ribs of the roadway below the RCP are 3.4 m and 5 m, respectively. The crest value of deviatoric stress reaches 10 MPa. As the distance between the panel and the RCP decreases, the shape of the high deviatoric stress area presents the evolution law from the “ellipse” of the roof → the “crescent” of two ribs → the “cochlea” of the tips of the ribs. (3) When the mining of the underlying panel is 10 m, 0 m, or − 10 m away from the RCP (without passing through the RCP). The crest value of deviatoric stress within 5–10 m in advance of the roadway increases in turn. However, the peak value is significantly reduced when it is − 20 m away from the RCP (through the RCP). The crest value of deviatoric stress of two ribs decreases in turn along the panel rib → section coal pillar rib → solid coal rib. Based on this, the underlying 45 m of the RCP is divided into area I (10 m), area II (overlapping area 20 m), and area III (15 m) based on the degree of disturbance. And propose the technical scheme of asymmetric combined control in different zones by using asymmetric channel steel truss anchor cable for the top-ribs of areas I and III, and top-ribs asymmetric channel steel truss anchor cable + door-type support in area II. On-site project practice shows that the partitioned control technology successfully resisted the roadway instability and failure caused by the dynamic-static superimposed stress disturbance under the RCP and realized the primary support of the sectional coal roadway. The conclusion provides technical support and scheme design for the partitioning support of roadways under similar “+” type cross‐panels.

## Introduction

**Figure. 1:**
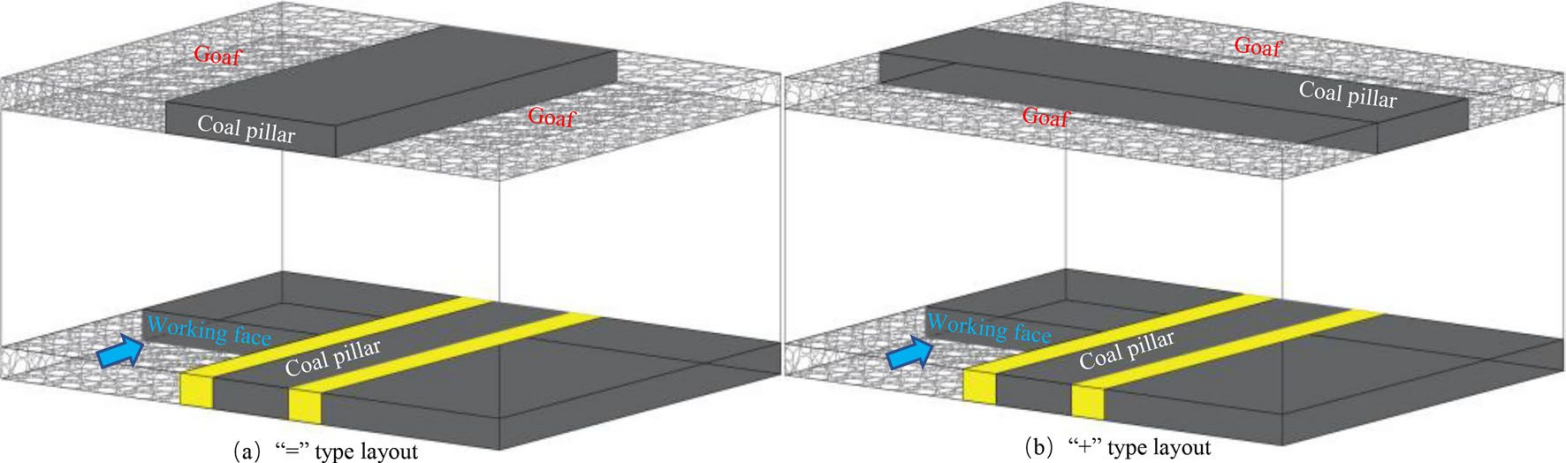
Different spatial layout types of coal pillars

CDCS is distributed far and wide. However, most of the upper coal seams in CDCS have been mined out, facing many complex conditions in the mining of the lower coal seam, and the complex problems of stability maintenance in the mining roadway are increasing day by day. Regarding CDCS mining, the reasonable width of the coal pillar and the roadway support technology are the current research hotspots of great significance to the reasonable and efficient recovery and exploitation of coal energy. The exploitation of coal energy has entered a brand-new stage^1^. The current arrangement of CDCS can be divided into two types, as illustrated in Fig. 1a,b. That is to say, the spatial arrangement of the two coal seams' working faces is of “=” type and “+” type; most of the current research aims at the “=” type layout.

Numerous academics have done extensive research on the “=” spatial arrangement of CDCS. Chen et al.^2^ investigated the elastic core width and the evolution of damage patterns from asymmetric to symmetric under the conditions of CDCS mining with different widths of protection pillars and proposed the corresponding asymmetric support scheme. Chen et al.^3^ investigated the deviatoric stress regularity of surrounding rock under dynamic pressure in multi-seam mining and the damage depth of the plastic zone. Ru et al.^4^ pinpointed the roof’s creep failure of the underlying mining roadway caused by the stress transfer of the RCP under shallow burying and CDCS, putting forward the supporting measures for the roof. Yang et al.^5^ elaborate on the approach of weakening and relieving pressure by high-pressure water jets to address the issue of high ground pressure under the RCP in CDCS, which cuts off the transmission route of overlying loads to a certain extent. Yang et al.^6^ simulated the effective anchoring depth of bolts under different intervals in CDCS by theoretical calculation and experiment. They formulated the corresponding roof bolts supporting the scheme to be applied. Zhang et al.^7^, focusing on large deformation of the surrounding rock with impact tendency under the hard roof of the CDCS, elaborate the approach of interleaving the panel and setting the narrow protective coal pillar, effectively mitigating the deformation of the roadway. Wu et al.^8^ analyzed the stress law under coal pillar according to the problems of roof breakage and large roadway deformation caused by the geological factor of the CDCS; the reasonable support scheme under different interleaving distance arrangements is put forward for the first time. He et al.^9^ studied the stress law of surrounding rock and the destroyed form of the plastic zone in the retracement channel. Aiming at the phenomenon of multiple disturbed mine pressures in the roadway under the CDCS, the zoning treatment approach of “High-pressure water jet + asymmetric high-strength cable-beam net + three-hole anchor cable” is put forward. Zhang et al.^10^ took the stress space–time evolution law of stress of the simultaneous mining of multiple working faces of CDCS in the Nantun coal mine as the analysis object, analyzed the three subareas of stress evolution, and noted that the roadway should be placed in the low-stress area to improvement the reliability of the roadway. Shang et al.^11^ found that the roof pressure in the area of the elastic core of RCP is high, which influences mining in the lower coal seam. Xiong et al.^12,13^ verified the bad self-stability of the surrounding rock, which is easily disturbed and broken by engineering. The repairing scheme of long bolt + high strength anchor Cable + U-shaped steel + grouting is put forward to prevent the roadway from losing stability. Sun et al.^14^ utilized the half-plane theory and Mathematica software to analyze the principal stress difference evolution law of coal pillar floor in CDCS and, based on this, put forward the reasonable control method of the roadway and reduced the failure of the underlying roadways. Wei et al.^15^ obtained the microscopic pore characteristics of remolded loess and undisturbed loess at different static ages by using electron microscope observation and nuclear magnetic resonance test. They found that thixotropy increased the cohesion and friction of soil structure, which explained the increased thixotropic strength at the macro scale. Wang et al.^16^ studied the damage process and characteristics of granite samples treated with liquid nitrogen at 300 °C by using a compact scanning electron microscope and nonmetallic ultrasonic testing analyzer. They found that liquid nitrogen cooling treatment can reduce the mechanical properties of rocks, and the damage is more serious at high temperatures. Wang et al.^17^ studied the fracture characteristics, acoustic emission characteristics, and rock burst trend of granite under different cycles of liquid nitrogen cooling. The results show that it promotes the formation of internal cracks. Liu et al.^18^ studied the effects of particle size of coal and gangue, microwave radiation time, and microwave frequency on the thermal sensitivity of microwave radiation. They proved the feasibility of the method of identifying the activity of coal gangue by microwave radiation combined with infrared detection. Lyu et al.^19^ studied the feasibility of building a pumped storage power station from abandoned mines in combination with the geological conditions in Shitai Mine, Anhui Province, China, and successfully implemented it in Shitai Mine. Lyu et al.^20^ made the coal pillar artificial dam assembly and conducted uniaxial compression tests with different wetting cycles. It was found that the wetting cycles were positively related to the damage to the sample. In the wetting process, ettringite and xonotlite were formed in concrete, and C–S–H groups were to invade the coal pillar. Lyu et al.^21^ clarified the transformation direction of carbon sequestration in abandoned mines by understanding the development direction of carbon sequestration in abandoned mines. They put forward several suggestions for the transformation of coal enterprises under the goal of carbon neutrality. Cheng et al.^22^, to acquire the features of the roof movement and the evolution of mining stress, obtained that the roof strata are multi-strata cooperative movement and formulated high-resistance support for superimposed mining. He et al.^23^, through the mechanism of the impact of stress concentration in the coal pillar at the roof of the CDCS, the research results show that the roof is loaded with the RCP in the mining, it is easy to break in advance, which will affect the safety mining of the panel. Lv et al.^24^ studied the failure properties in the coupling superimposed disturbance zone of the retracement roadway under the close-distance RCP in the Yanzishan coal mine. They obtained the appropriate location of the retracement roadway to avoid the large deformation of roadways under high-stress disturbance. Zhao et al.^25–27^ systematically studied the damage mechanism of the RCP floor in CDCS and the damage pattern of the roadway under mining stress superimposed disturbance and put forward the corresponding support technology. Chen et al.^28,29^ have a detailed study of the stope rock plate structure fracture, and through deviatoric stress, J2, and other multi-index combined methods^30,31^ analysis of the roadway surrounding rock stress state and failure. Xie et al.^32^, through the deep large-scale variable cross-section roadway support complex and large damage engineering characteristics, proposed the integrated cooperative stability control technology of more than 7 m reinforcement compression arch. Under the condition of continuous large deformation of deep mine roadway, a suitable control method of “External anchor-internal unloading”^33^ is put forward, which solve the issue. Li et al.^34^ derived the overlying strata stress field formula by the Schwarz alternating method in CDCS repeated mining. It concluded that mining the lower coal seam will cause secondary development of the fracture zone in the overlying strata. Du et al.^35^ used UDEC software to analyze the stability of the lower coal pillar in the CDCS; the evolution characteristics from goaf to section pillar and then to goaf structure are revealed. Zhang et al.^36^ studied the influence mechanism of RCP in room and pillar mining on the underlying working face. They revealed that the inverted trapezoid collapse occurred above the coal pillar. Li et al.^37^, through the CDCS, repeated mining broken roof easy to appear section leakage, elaborated on the cantilever hinged form and gave a suitable support work resistance design. Liu et al.^38^ analyzed the stability of the stope roof by studying the problem of complex maintenance and multiple disturbances of gob-side entry under CDCS. They illustrated the critical area of roof support under goaf. Sinha et al.^39^ used the self-defined constitutive model to predict the yield failure of coal pillars relatively accurately and, to a certain extent, can solve the roadway coal pillar ribs collapse and cause the accident. Ning et al.^40^ studied the mechanical mechanism and evolutionary mechanism of overlying coal seam crushing, established a mechanical model of overlying strata secondary “Activation,” and improved the accuracy of fracture zone height prediction. Zhang et al.^41^ calculated the upper coal seam floor’s failure depth using the plastic slip line field theory and verified it using numerical simulation, and the proper position and support parameters of gob-side entry can be determined. Liu et al.^42^ constructed a non-uniform superimposed stress model of a coal pillar based on energy buildup in the coal pillar, revealed the nature of deformation of the underlying roadway, and suggested that the underlying roadway ought to be placed in the area of pressure relief or shear slip.

The above scholars have done much research on the destruction range of upper seam floor and proper position and support of underlying roadway in CDCS safe mining and have made a specific contribution to the development of CDCS mine resources. However, the existing research is only for the “=” panel type, as shown in Fig. 1a. Therefore, the above research results are not entirely applicable to the “+” type spatial cross-layout of close-distance coal seams, which is rarely studied. According to the research of other scholars, when the panel is arranged in “=” type, the roadway layout of the underlying coal seam can avoid the high-stress peak area. However, in the “+” type layout, the roadway in the lower coal seam must pass through the overlying RCP and the roadway is seriously damaged and deformed in the superimposed high-stress area when the panel passes through the RCP. Due to the unclear failure mechanism of the surrounding rock, it is impossible to form an effective and reasonable support scheme. Therefore, this paper will study how to effectively support the roadway under the RCP in such conditions from various methods, such as field measurement, numerical simulation, and indoor testing. This research not only supplements the lack of existing research results of lower position roadway support in the “+” type panel layout, but also provides technical reference for the support of similar roadways.

## Engineering background

### Geological situation

**Figure. 2:**
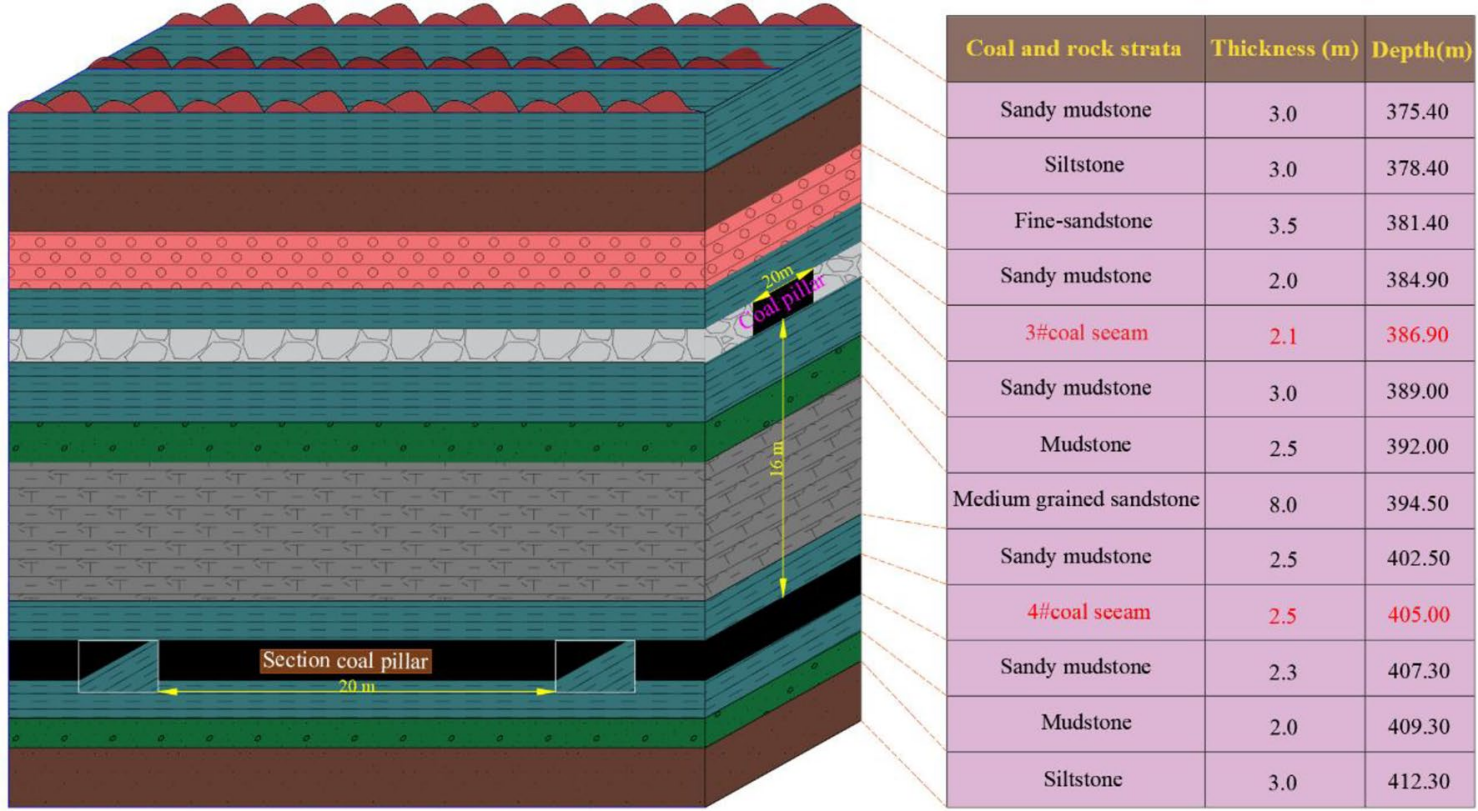
Coal and rock strata histogram.

**Figure. 3:**
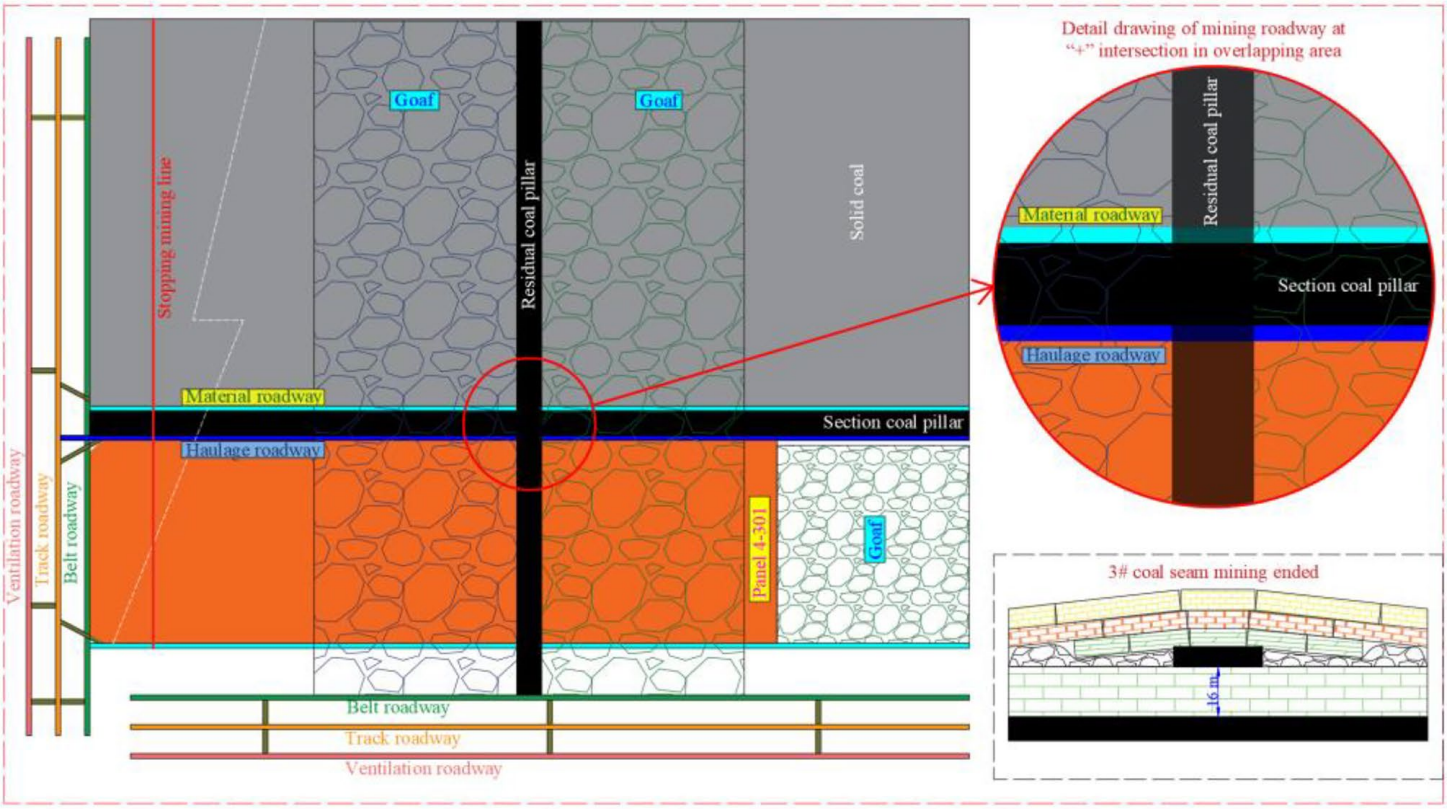
Arrangement diagram of panel 4-301.

A mine is located in Shanxi Province, China. The mineable coal seam of a mine is 3# and 4# two layers of coal, and the average thickness of 3# coal seam and 4# coal seam is 2.1 m and 2.5 m, respectively, and the average dip angle of two coal layers is 5°. The whole mine adopts the technology of single mining full-seam. There are four mining zones in the mine. The panel of 3# coal seam is arranged along the direction, and the panel of 4# coal seam is arranged along the tendency. The research object is situated in the third mining zone, in which the interlayer spacing between 3 and 4# coal seams is 16 m. The average buried depth of 4# coal seam is about 405 m. The lengths of the upper and lower coal seam panels are 200 m and 180 m, respectively. The width of the coal pillar in the upper and lower coal seam is 20 m. The coal and rock strata histogram is illustrated in Fig. 2:

The panel layout of 3# and 4# coal seams is spatial cross-type, which differs from the conventional CDCS mining method. The 3# coal seam in the mine has been mined out, leaving 20 m coal pillars, and the panel of 4# coal seam is directly below. The underlying panel will pass through the RCP, which will easily lead to safety problems such as roof caving and support failure. According to the site conditions, the 4–301 panel of lower coal seam will pass through the RCP. The arrangement diagram of panel 4–301 is illustrated in Fig. 3:

### Mine pressure appearance and damage of mining roadway in overlapping area

**Figure. 4:**
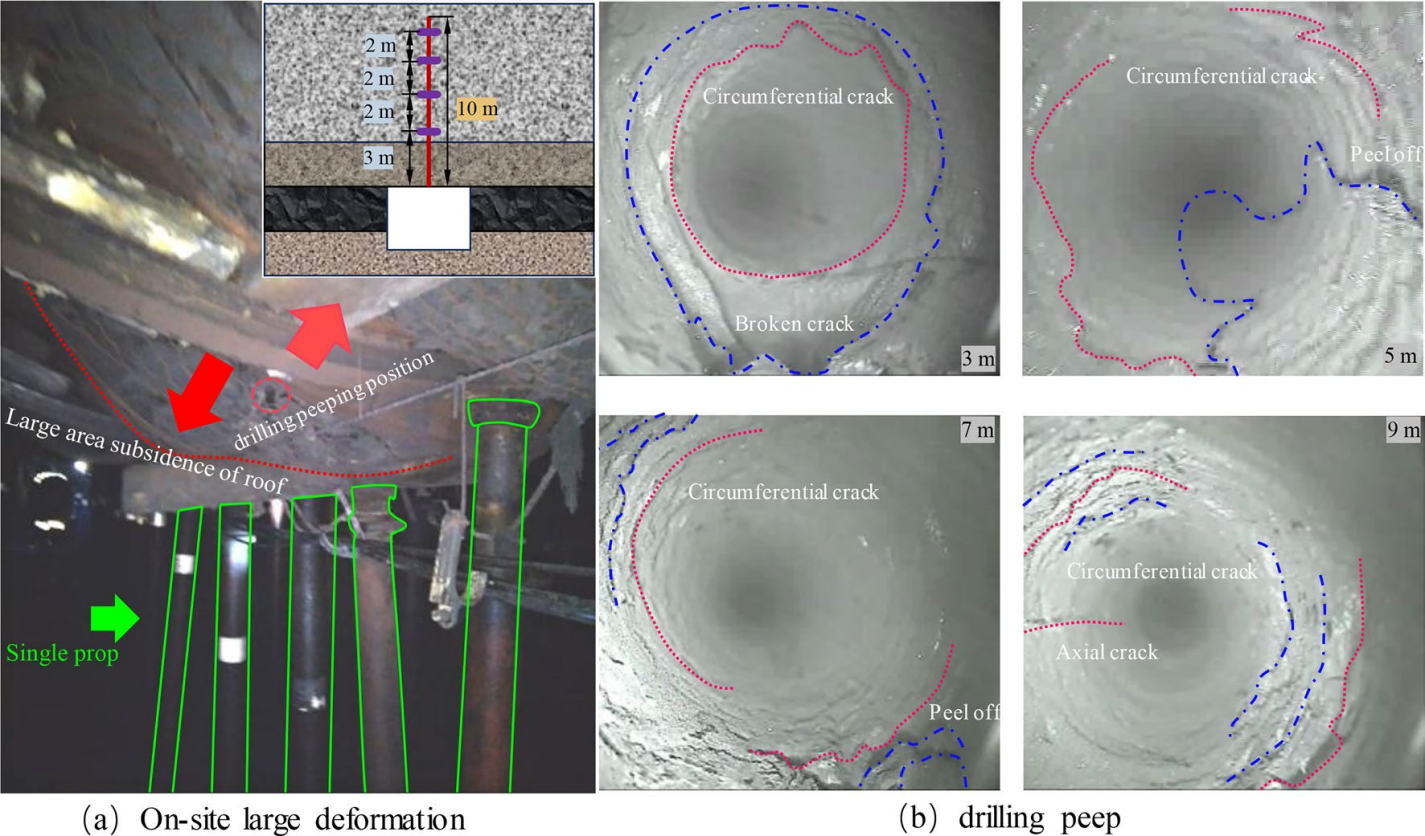
On-site damage and drilling peep.

The phenomenon of high ground pressure in mining roadways in the “+” cross area is obvious. The roof of the panel 4-205 in the same geological unit has large deformation in the overlapping area, and the on-site roof drilling peep is as follows:

As illustrated in Fig. 4a, the mining roadway roof in the overlapping area is sinking in a large area. The support means, such as a single column, are adopted in time, but the intense mine pressure can still not be controlled. The deformation of the mining roadway continues to increase, seriously affecting safety production. In Fig. 4b, the roof drilling peep is carried out within 9 m. The roadway roof rock in the overlapping area is significantly damaged, and the annular cracks and spalling are all over the whole peep depth.

## Numerical simulation analysis

Due to the surrounding rock destruction in the above overlapping areas, it is necessary to establish a numerical model for further analysis in combination with the site damage situation to provide some guidance for roadway support.

### Model establishment

**Figure. 5:**
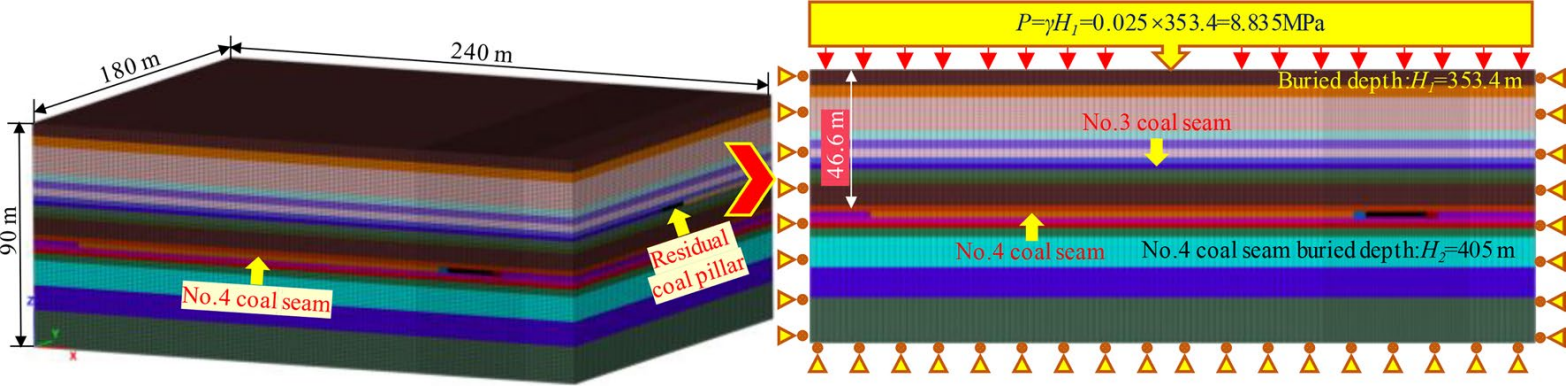
Establishment of three-dimensional numerical model.

The model is established to study the stress distribution of the roadway’s surrounding rock under the overlapping area of the RCP in a CDCS mine. Figure 5 illustrates the model size: 240 m × 180 m × 90 m. The boundary around and at the bottom of the model is fixed, and 8.835 MPa compressive stress is applied to simulate the generated load. The Mohr–Coulomb constitutive relationship is adopted.

The numerical simulation adopts the distributed excavation method to simulate the actual advance distance of the panel. The simulation sequence is the end of 3# coal seam mining → 4# coal seam roadway excavation → 4-301 panel mining. According to the numerical simulation results, this paper analyzes the plastic zone expansion form and deviatoric stress evolution law of surrounding rock before and after mining influence. A good and reliable support scheme is formulated in combination with the site. Through field sampling and laboratory specimen preparation, the author conducted the test and calculation to obtain the physical and mechanical parameters of coal rock, as shown in Table 1.

**Table 1:** Physical and mechanical parameters of coal seam and rock stratum.

Coal and rock stratum	Density/(kg m^−3^)	*K*/GPa	*G*/GPa	*c*/MPa	*σ*^*t*^*/*MPa	*φ*/°
Sandy mudstone	2631	5.16	4.23	1.98	1.63	30
Siltstone	2745	9.11	7.14	2.97	1.97	26
Fine-sandstone	2767	9.83	7.27	3.02	3.01	29
Coal seam	1344	2.33	1.63	0.91	0.37	23
Mudstone	2287	4.81	2.54	1.62	1.28	22
Medium grained sandstone	2690	9.43	6.23	4.37	3.46	29

Where *K* is bulk modulus, *G* is shear modulus, *c* is cohesion, *σ*^*t*^ is tensile strength, *φ* is internal friction angle.

### Measurement index of roadway surrounding rock failure

Based on the classical elastic–plastic theory and the rock soil elastic–plastic mechanics theory, the stress tensor can be decomposed into spherical stress tensor and deviatoric stress tensor, and the changing state of the object under the action of external force is divided into volume change and shape change. Different stress control objects change differently, which can be divided into elastic strain and elastic shear strain. It is generally believed that deviatoric stress is the kernel element for plastic deformation and failure of objects^43,44^.

1 $$ \sigma_{ij} = \sigma_{m} \delta_{ij} + S_{ij} $$

In the formula: $\sigma_{m}$ is the spherical stress tensor; $\delta_{ij}$ is Kronecker symbol; $Sij$ is the deviatoric stress tensor.

2 $$ \left( {\begin{array}{*{20}c} {\sigma 1} & 0 & 0 \\ 0 & {\sigma 2} & 0 \\ 0 & 0 & {\sigma 3} \\ \end{array} } \right) = \left( {\begin{array}{*{20}c} P & 0 & 0 \\ 0 & P & 0 \\ 0 & 0 & P \\ \end{array} } \right) + \left( {\begin{array}{*{20}c} {\sigma 1 - P} & 0 & 0 \\ 0 & {\sigma 2 - P} & 0 \\ 0 & 0 & {\sigma 3 - P} \\ \end{array} } \right) $$

In the formula, the first term on the right side of the equation is the spherical stress, and the expression is $P = (\sigma 1 + \sigma 2 + \sigma 3)/3$; The second term is deviatoric stress. Among them, $\sigma 1 - P$ also known as the maximum principal deviatoric stress *S*_1_, is the dominant force of deviatoric stress. It is generally believed that the maximum principal deviatoric stress is the deviatoric stress. The indicator used in this article to measure deformation and failure is the maximum principal deviatoric stress (referred to as deviatoric stress)^45,46^.

The deviatoric stress formula is as follows:

3 $$ S1 = \sigma 1 - \frac{1}{3}(\sigma 1 + \sigma 2 + \sigma 3) $$

According to formula (3), the deviatoric stress includes three principal stresses, and it is more intuitive to evaluate the rock state. Therefore, the deviatoric stress is used as the evaluation index for analysis.

## Analysis of simulation results

### Distribution and evolution law of deviatoric stress on the floor of the RCP

**Figure. 6:**
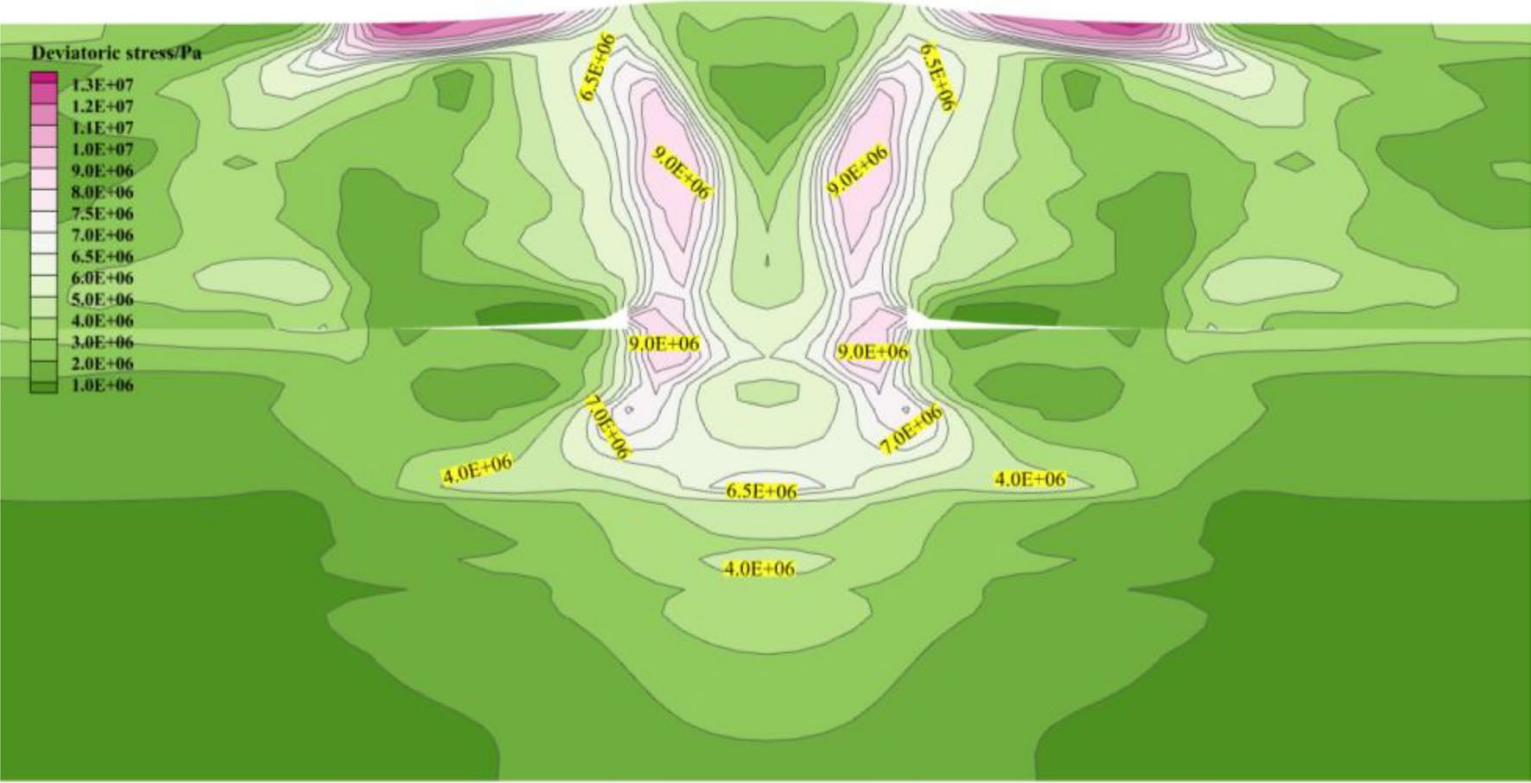
Distribution of deviatoric stress in RCP floor.

As illustrated in Fig. 6, after the completion of the mining of 3# coal seam, the deviatoric stress of the RCP floor diffuses symmetrically towards the floor and decays accordingly. The deviatoric stress below the RCP floor forms an elliptical peak circle. The peak deviatoric stress of RCP appears near the goaf side of the floor, with a peak value of 9.0 MPa, which then decreases to 6.3 MPa, forming a continuous secondary peak belt of deviatoric stress.

Through the above analysis, we can know that there is a peak belt of deviatoric stress within a specific range below the floor of the RCP, which is in a critical failure state. Therefore, different distances below the floor are selected in the numerical model for monitoring the deviatoric stress values to clarify the trend of deviatoric stress attenuation along the floor of the RCP.

**Figure. 7:**
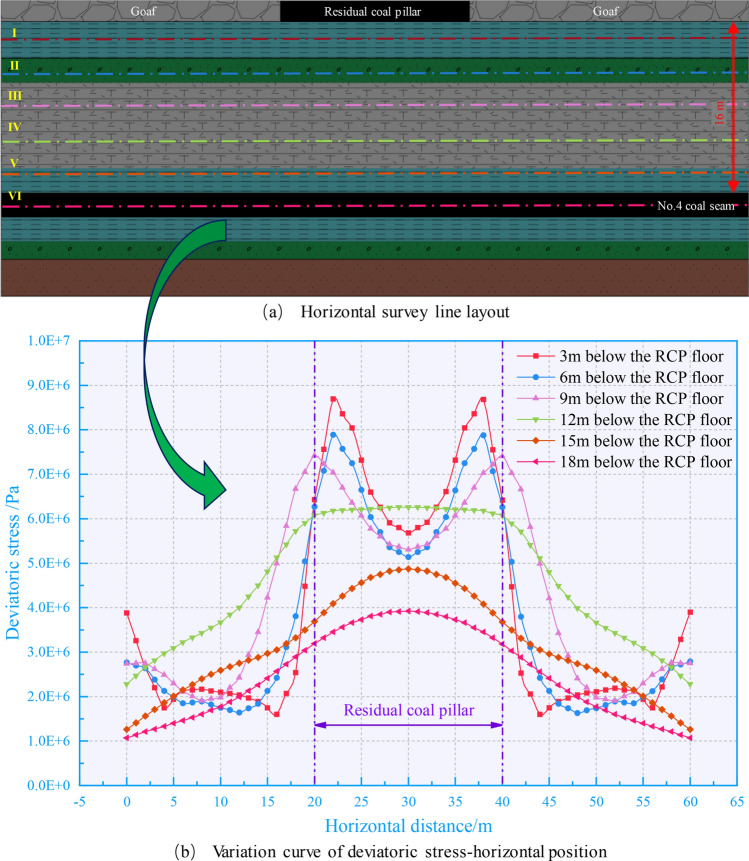
Different horizontal deviatoric stress curves of residual coal pillar floor.

As illustrated in Fig. 7, a measuring line is arranged every 3 m below the RCP to monitor. Within 9 m below the RCP, the deviatoric stress shows a double peak shape, decreasing the peak value from 8.6 to 7.4 MPa. Subsequently, the deviatoric stress shape changes from double peak to single peak. The peak value decays to 3.9 MPa when the measuring point is in the 4# coal seam directly below the RCP. In summary, the coal directly below the RCP is more prone to plastic failure after being disturbed.

### Evolution law of plastic zone failure and deviatoric stress in mining roadway

#### Evolution of deviatoric stress and plastic zone range after roadway excavation

**Figure. 8:**
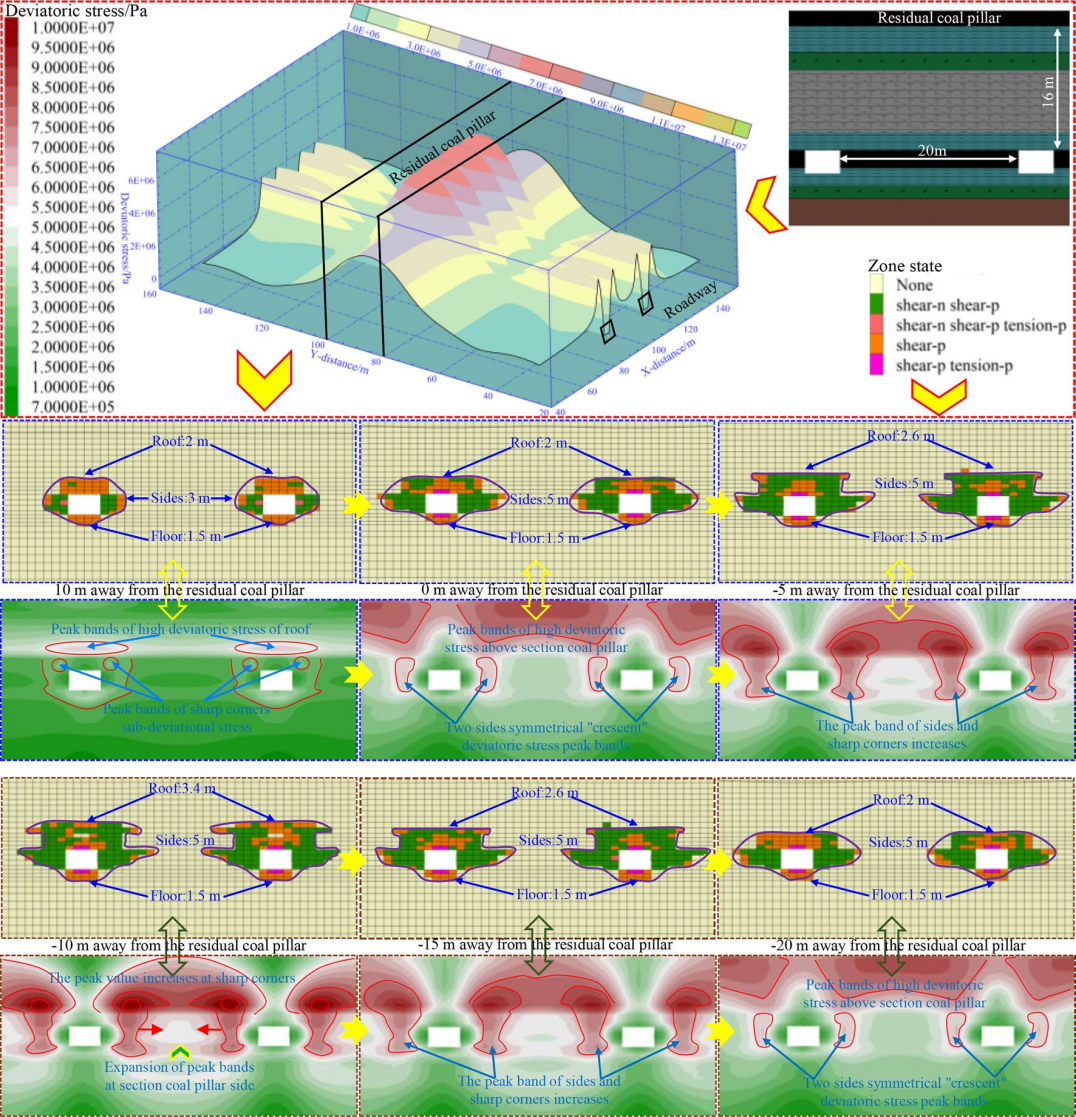
Plastic zone and deviatoric stress distribution after roadway excavation.

As illustrated in Fig. 8, the deviatoric stress on two ribs of the mining roadway located under the RCP is obviously higher than that below the goaf, with a peak value of over 7.0 MPa. Moreover, the peak deviatoric stress on the section coal pillar rib is higher than that on the solid coal rib. Therefore, it can be inferred that the two ribs of the roadway in the overlap area below the RCP are more prone to failure when disturbed. Select the plastic zone and deviatoric stress of the surrounding rock at different distances from the RCP after excavation of the mining roadway for analysis:

1. When the distance from the RCP is 10 m, the widths of the plastic zone on the two ribs, roof, and floor of the mining roadway are 3 m, 2 m, and 1.5 m, respectively; The roof has an “elliptical” shaped peak stress belt, and there is a minor stress concentration area at the sharp corners of the roof, with a concentrated stress value of 5.6 MPa. The degree of stress concentration on both sides is low, and the critical failure area of the mining roadway is at the roof and sharp corners.
2. When the distance from the RCP is 0 m (reaching one side of the RCP), the width of the plastic zone on both ribs increases to 5 m. The concentration of deviatoric stress on both ribs of the roadway increases, forming a flat “crescent” shape with deviatoric stress of 6.0 MPa. The peak deviatoric stress at the roof’s sharp corner reaches above 7.0 MPa. The range of deviatoric stress concentration above the section coal pillar increases. The peak deviatoric stress reaches 8.4 MPa, and the plastic zone further expands towards the two ribs.
3. When the distance from the RCP is − 5 m, the depth of the plastic zone on the roof of the mining roadway increases to 2.6 m, and the peak stress belts on both ribs and the sharp corner stress concentration zone expand and merge, forming a “cochlear” shaped stress concentration zone with the peak value of 9.2 MPa. Moreover, the stress concentration zone moves downward, and the two ribs and roof sharp corners of the mining roadway are more prone to plastic failure when disturbed.
4. When the distance from the RCP is − 10 m (below the RCP), the plastic zone of the mining roadway roof increases to 3.4 m, and the degree of stress concentration at the roof’s sharp corner is evident, reaching a peak of 10 MPa. Here, the plastic zone of the roadway and the degree of stress concentration reach a peak, and the mining roadway is more easily damaged.
5. When the distance from the RCP is − 15 m or − 20 m (on the other side of the RCP), the plastic zone and deviatoric stress evolution law of the mining roadway are similar to that when it is − 5 m or 0 m from the RCP. However, due to the paper’s length, there will be no further elaboration here.

In summary, the failure situation and deviatoric stress evolution law of the mining roadway below the RCP are approximately symmetrically distributed, and the mining roadway directly below the RCP is more prone to failure.

#### Evolution of deviatoric stress and plastic zone range during panel mining cross RCP

**Figure. 9:**
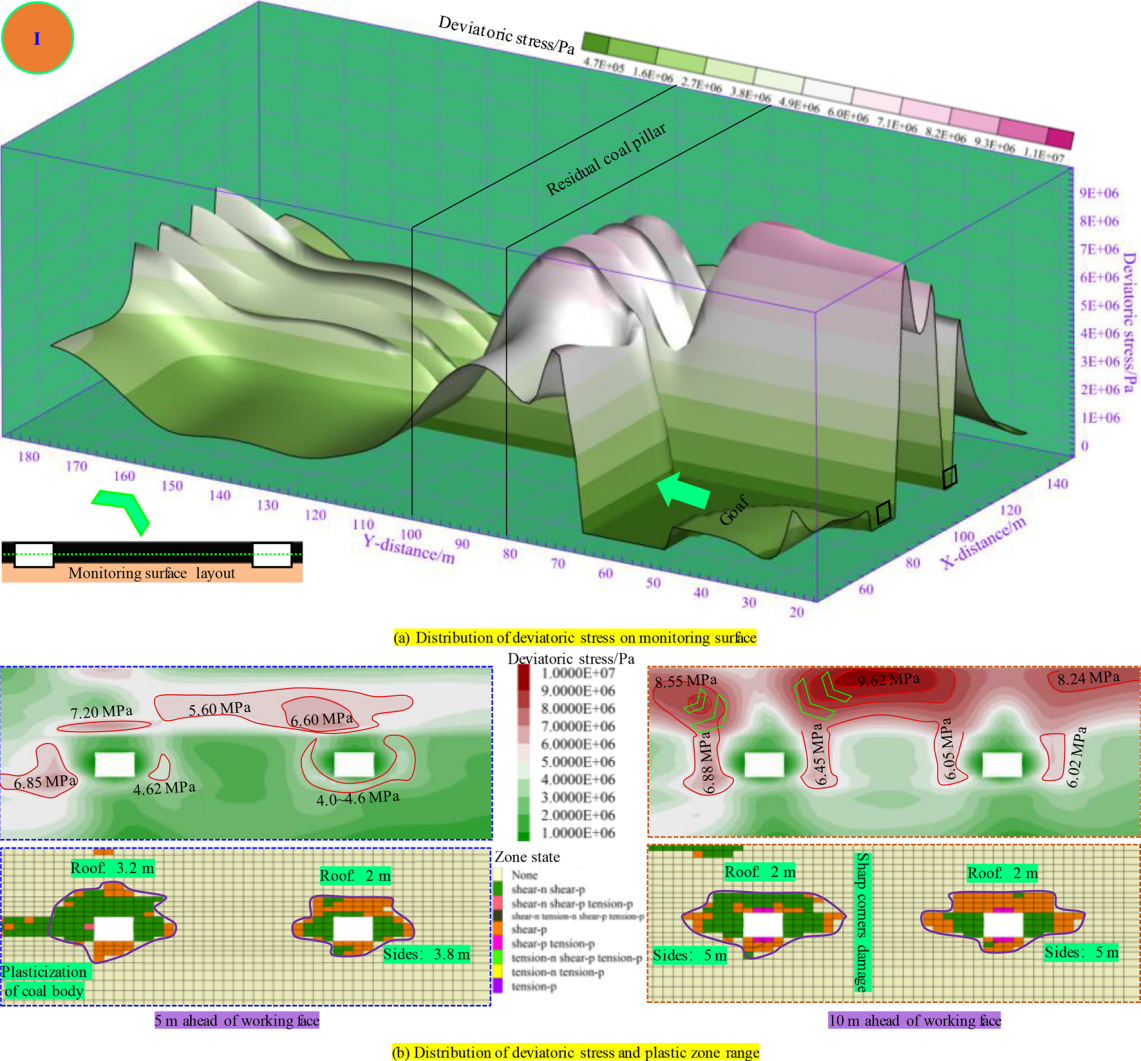
Distribution of plastic zone and deviatoric stress during mining 70 m in panel.

The foregoing research can preliminarily distinguish the plastic zone and deviatoric stress evolution law of the excavated mining roadway below the RCP. However, the failure of the roadway and the distribution law of deviatoric stress caused by dynamic pressure disturbance during the panel mining is not yet apparent, and detailed analysis is still needed for different stages of the mining process:

As illustrated in Fig. 9, when the panel is mined to the distance of 10 m from the RCP, due to the superposition of advanced disturbance stress, the deviatoric stress value on the panel side of the mining roadway is high, with the peak deviatoric stress of over 7.0 MPa. The mining roadway’s overall deviatoric stress and plastic zone range 5 m in advance of the panel are small. The deviatoric stress on the adjacent panel side is relatively large. The plasticization extent of the adjacent panel side of the roadway is high, with a depth of 3.2 m in the roof plastic zone. In comparison, the plastic zone depth of the roof and two sides of the right roadway are 2 m and 3.8 m, respectively. At 10 m in advance of the panel, the mining roadway (when reaching one side of the RCP) is affected by the mining stress and the RCP. The distribution of the stress value on the mining roadway roof is as follows: the RCP below (9.62 MPa) is higher than the working face side (8.55 MPa). While on both ribs of the roadway, the RCP rib is lower than the working face rib. At this point, the roof plastic zone is relatively small (2 m), while both sides increase to 5 m, and damage begins at the sharp corners of the mining roadway roof.

**Figure. 10:**
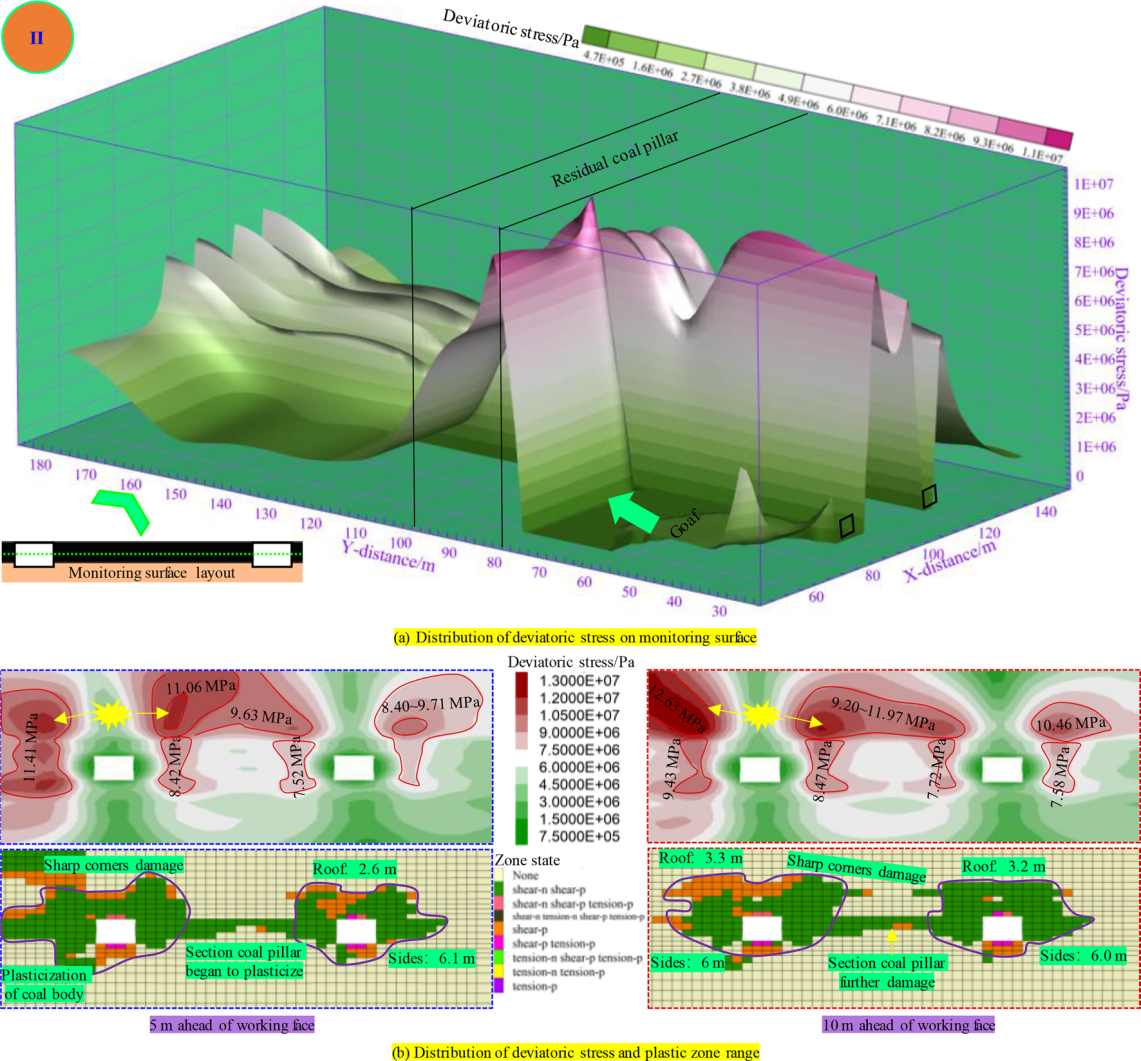
Distribution of plastic zone and deviatoric stress during mining 80 m in panel.

As illustrated in Fig. 10, when the distance from the RCP is 0 m, the deviatoric stress in advance of the panel increases to 11 MPa, and the plastic damage range increases accordingly. The plastic penetrate damage began to occur in the overlapping area below RCP. The deviatoric stress of the panel rib of the underlying mining roadway at 5 m in front of the panel reaches 11.4 MPa, and the peak point is located at the sharp corner. The plastic zone extends to the sharp corner. The peak zone of deviatoric stress far from the panel rib is “cochlea”. Due to mining, the overall deviatoric stress distribution is higher on the panel rib than on the coal pillar rib. The plastic failure depth of the roof increased to 2.6 m, and the plastic failure depth of the two ribs reached 6.1 m. The peak value of deviatoric stress at the roof corner of the panel rib 10 m in advance of the panel (the center of the RCP) reaches 12.6 MPa. The peak range of deviatoric stress at the roof of the section coal pillar increases, and the deviatoric stress value is between 9.20 and 11.97 MPa. The overall peak point distribution is: working face rib → section coal pillar rib → solid coal rib, and the peak value decreases. The roof plastic damage depth is above 3.2 m, and the damage range increased. The sharp corners on the coal pillar are damaged, and the damage depth of the two ribs is 6.0 m.

**Figure. 11:**
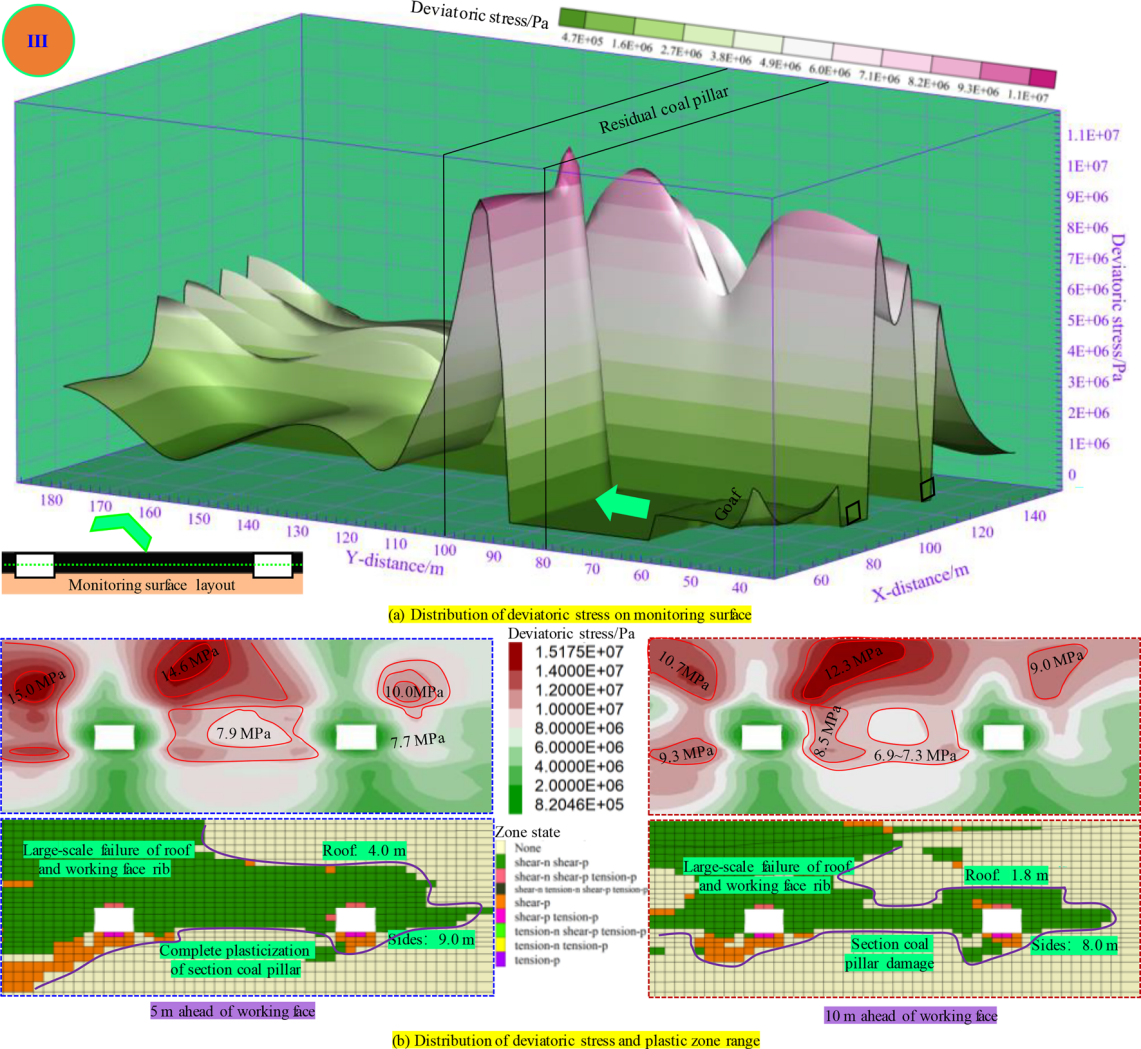
Distribution of plastic zone and deviatoric stress during mining 90 m in panel.

As illustrated in Fig. 11, when the panel is − 10 m away from the RCP (the middle part of the RCP), the peak value of the deviatoric stress of the two ribs of the underlying haulage roadway (panel rib) reaches more than 10 MPa. The maximum deviatoric stress of the roof corner of the haulage roadway at 5 m in advance of the panel reaches 15.0 MPa, and the overall deviatoric stress of the section coal pillar reaches more than 7.9 MPa. At this time, the underlying mining roadway was destroyed in a big area, all the section coal pillars were plasticized, and the bearing capacity of coal and rock mass was reduced, seriously affecting the stability of the mining roadway. The peak value of deviatoric stress at the roof sharp corner of the section coal pillar at 10 m in front of the panel reaches 12.3 MPa. At this time, the haulage roadway’s roof is still damaged in a large area, and the coal pillar is plasticized, which makes it difficult to keep the safety of the mining roadway.

**Figure. 12:**
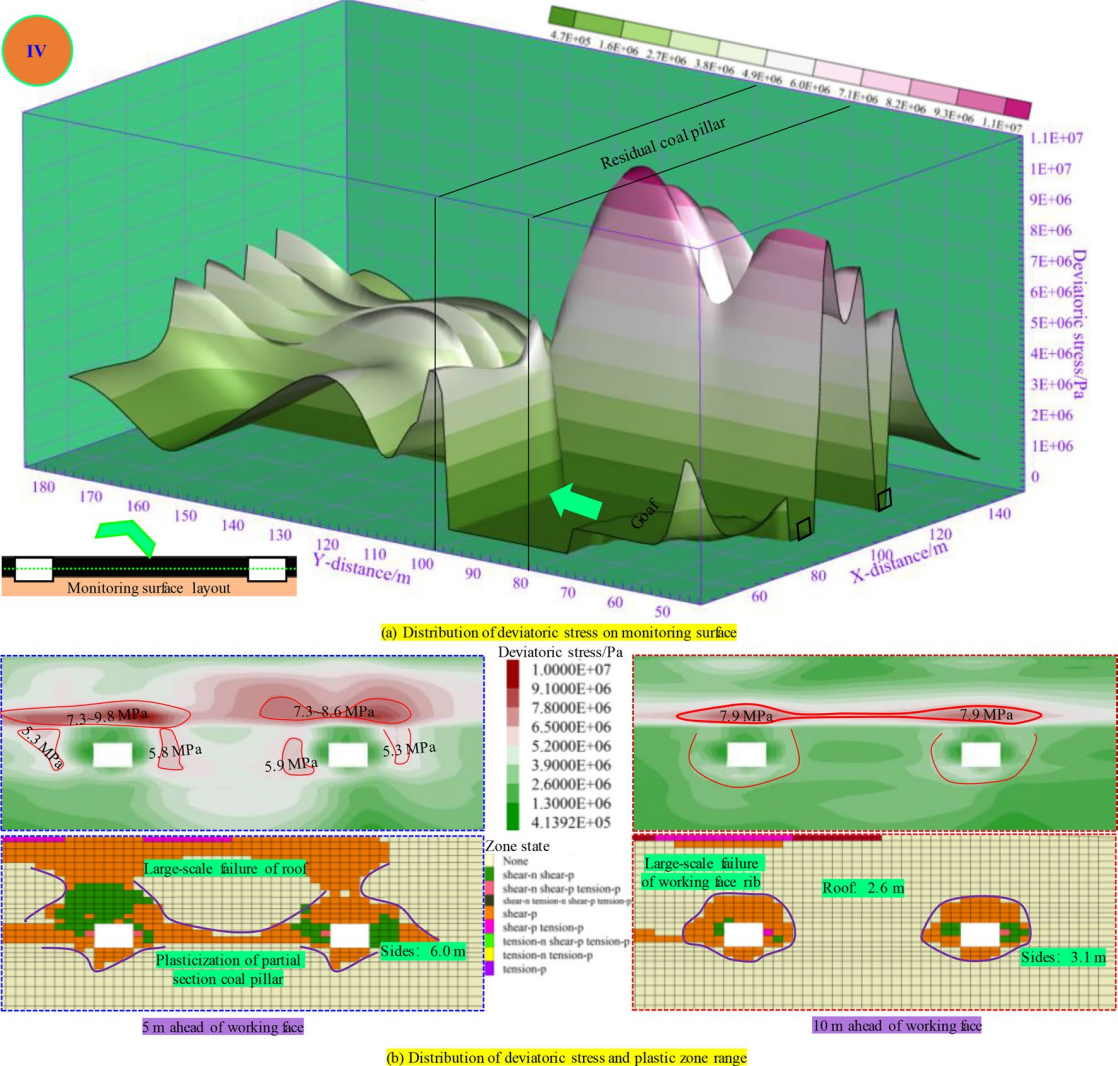
Distribution of plastic zone and deviatoric stress during mining 100 m in panel.

As illustrated in Fig. 12, when the panel passes across the RCP, the deviatoric stress in front of the panel decreases, and the peak point is below the RCP. The peak value of deviatoric stress in the roadway roof within 5 m in advance of the panel is above 7.3 MPa. At this time, plastic failure occurred at a certain distance above the coal pillar, and the roof failure range of the mining roadway was extensive, which made it difficult to maintain stability. The plastic zone depth of the two ribs was above 6.0 m. The peak value zone of deviatoric stress 10 m in front of the panel is located on the mining roadway roof, which is easily damaged by further disturbance. The failure range of the panel rib is extensive, and the roof plastic damage depth is 2.6 m, while the two ribs are 3.1 m. When the panel passes through the RCP, the value of deviatoric stress is reduced. However, they are still disturbed and easily damaged, so the support in this area still needs to be paid attention to.

**Figure 13:**
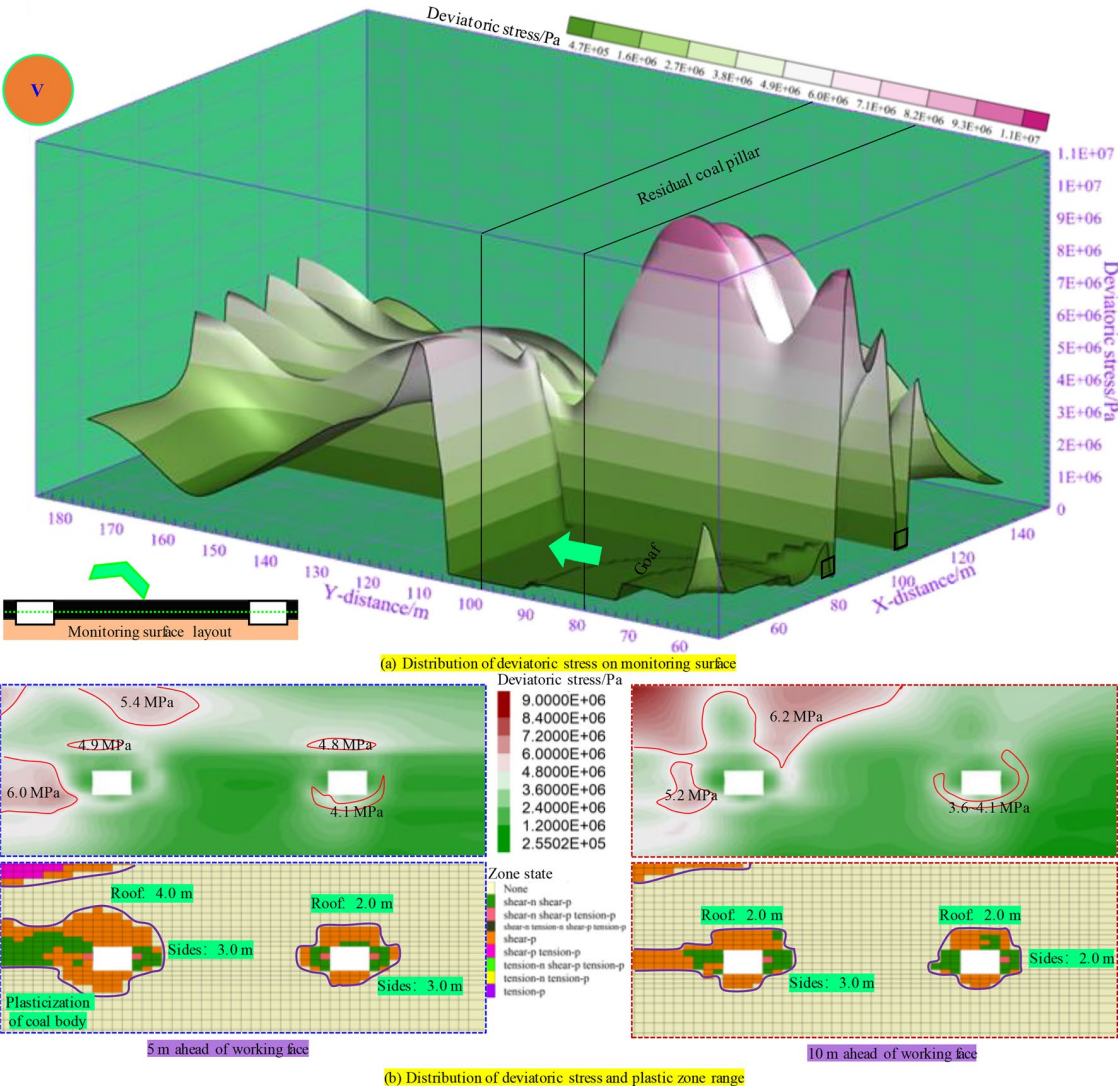
Distribution of plastic zone and deviatoric stress during mining 110 m in panel.

As illustrated in Fig. 13, when the panel passes across the RCP for 10 m, the deviatoric stress in front of the panel is significantly lower than that under the RCP. The peak of the deviatoric stress in the roof of the mining roadway within 5 m in front of the panel is reduced to 4.8–4.9 MPa, which is less than the peak of the deviatoric stress in the roadway working face side. The range of the roadway plastic zone is reduced, and the range of the roof plastic zone is 4 m, and the two sides are 3 m. It shows that although the underlying mining roadway is still affected by the overlying RCP, its influence on the roof of the underlying mining roadway begins to decrease, and the support in the 5 m area in front still needs attention. At 10 m in front of the panel, the disturbance of mining stress in the roadway is greatly reduced, and the plastic zone of the working face side of the roadway is greatly reduced. The peak zone of the roof of the mining roadway almost disappeared, and the deviatoric stress shifted to the deep. The peak value of deviatoric stress on the roadway side was reduced to 5.2 MPa, the value of deviatoric stress around the roadway returned to normal, and the influence of overlying RCP was eliminated.

**Figure. 14:**
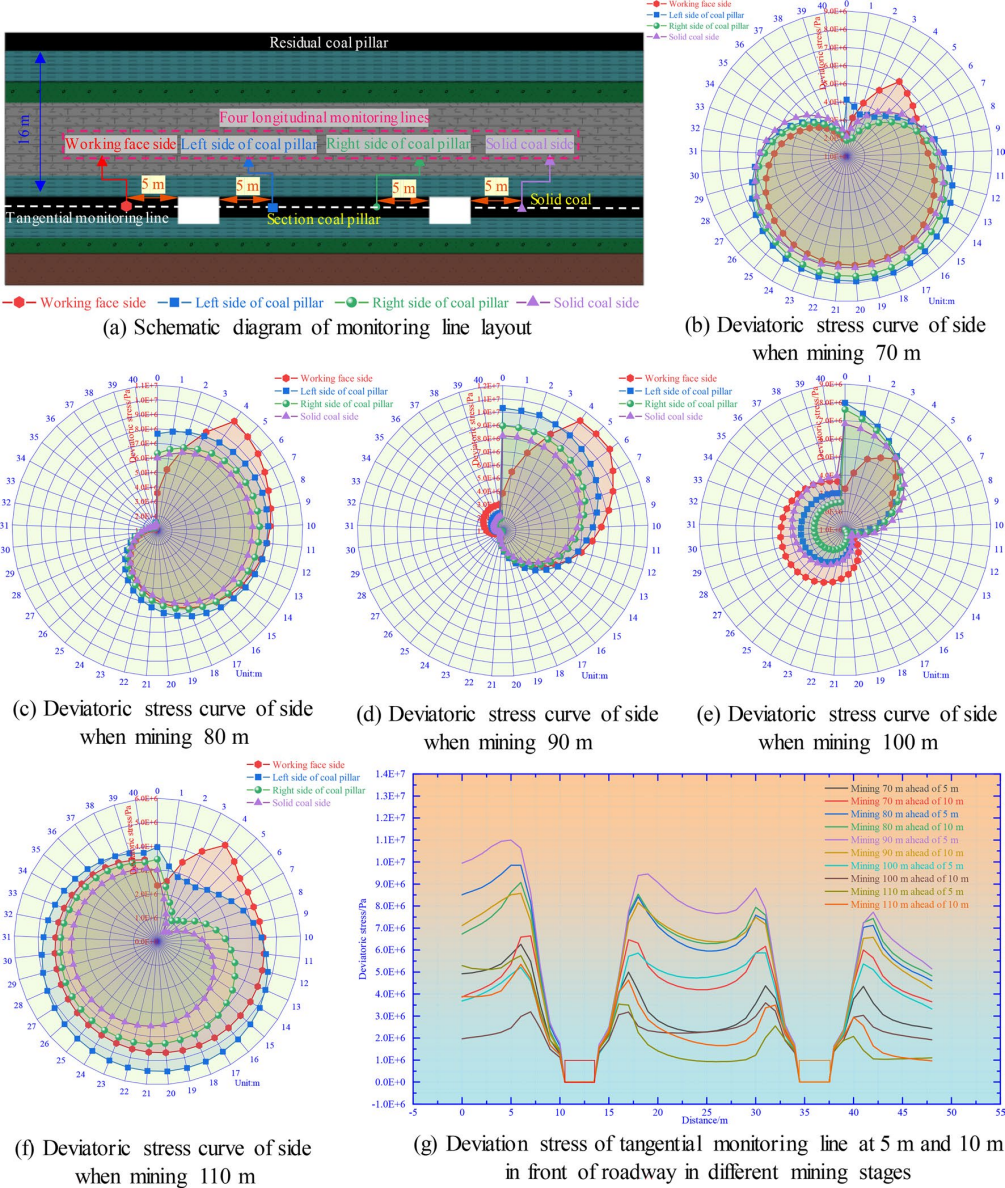
Distribution of deviatoric stress in two sides of roadway and different distances ahead in different mining stages.

As illustrated in Fig. 14, based on the analysis of Figs. 9, 10, 11, 12 and 13, the deviatoric stress in five different stages of mining is further analyzed: Fig. 14a is a schematic diagram of the arrangement of longitudinal monitoring lines and tangential monitoring lines. The longitudinal monitoring line monitors the distribution of deviatoric stress along the axial direction of the roadway in the range of 40 m in front of the panel, and the monitoring data are shown in Fig. 14b–f. The tangential monitoring line monitors the distribution of deviatoric stress in the tangential direction at 5 m and 10 m in front of the panel, and the monitoring data is shown in Fig. 14g. According to the obtained data, the specific analysis is as follows:

1. When the distance from the RCP is 10 m, the distribution trend and numerical value of the deviatoric stress within 40 m in advance of the rib of the underlying mining roadway are close, and the deviatoric stress is more significant only within 5 m in advance of the panel.
2. When the distance from the RCP is 0 m, the numerical value of the deviatoric stress within 40 m in advance of the rib of the underlying mining roadway is the panel rib > the left rib of the section pillar > the right rib of the section pillar > the solid coal rib, and the deviatoric stress within 15 m in front of the panel is above 8.0 MPa, and then gradually decline.
3. When the distance from the RCP is − 10 m, the peak value of deviatoric stress within 10 m in advance of the rib of the underlying roadway is high, which badly affects roadway safety.
4. When the panel is mined to a distance of − 20 m from the RCP (pass through the RCP), the value of the deviatoric stress within 40 m in advance of the rib of the underlying mining roadway is significantly reduced. The deviatoric stress is higher only in the range of 10 m front, which is easily further damaged by dynamic pressure.
5. When the panel is mined to a distance of − 30 m from the RCP (10 m through the RCP), within 5 m ahead, the deviatoric stress values of the coal pillar side and solid coal side of the roadway first decrease and then increase. However, compared with the last mining stage, the overall value of deviatoric stress decreased. The influence of the RCP on the roadway decreases, mainly due to the mining of the panel. In the range of 5–10 m in front of the roadway, the value of deviatoric stress began to rise, and the bearing capacity of the surrounding rock gradually recovered. The value of deviatoric stress outside 10 m in front of the roadway tends to be stable, and the difference value is small. At this time, the bearing capacity of the roadway surrounding rock is restored, and the influence of RCP has basically been eliminated.

To sum up, the deviatoric stress in the 5–10 m in front of the mining roadway is high, easily disturbed, and further damaged. In particular, after the panel passes through the RCP for 10 m, it is still slightly affected by the RCP only within 5 m ahead, so it is only necessary to pay attention to the roadway support within this range. The variation trend of deviatoric stress gradually decreases along the adjacent panel rib, but the coal pillar is easily damaged due to high deviatoric stress. At the beginning of the support design, the high deviatoric stress concentration caused by the influence of dynamic pressure in the overlapping area below the RCP should be considered to avoid further damage.

## Principle and design of asymmetric partition support

Based on previous analysis, due to the superposition of the advanced supporting stress and the concentrated stress of the RCP, the 20 m mining roadway in the overlapping area under the RCP is seriously damaged. When the panel is 10 m away from the RCP, the roadway in front will be affected by the more serious superimposed stress. Although the damage situation is better than the overlapping area under the RCP, it cannot be easily ignored. However, after the panel passes through the RCP for 10 m, only the roadway within 5 m in front is still slightly affected by the RCP, so the support strength should also be improved within 15 m in front after passing through the RCP. Accordingly, a specific area under the RCP is defined and provides the support scheme design.

### Partition definition

**Figure. 15:**
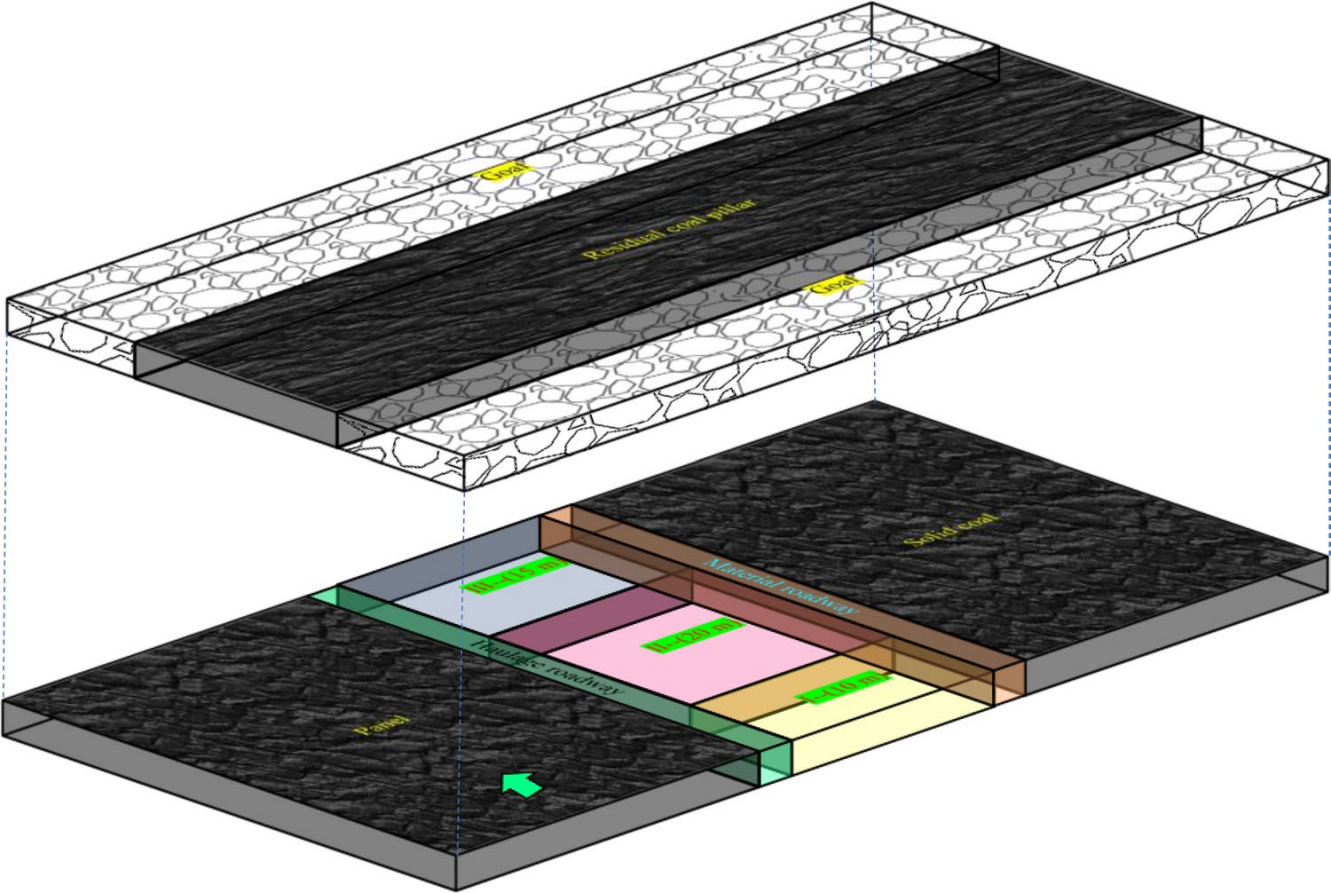
Delimitation of partition support.

As illustrated in Fig. 15, dividing the coverage of 45 m under the RCP into Area I (10 m), Area II (20 m), and Area III (15 m). Areas I and III are transitional areas on both sides of the RCP. Area II is the overlapping area under the RCP, the most severe area of damage. Due to the influence of different degrees of dynamic-static superimposed stress disturbance in the three areas under the RCP, the surrounding rock shows regional differential impairment, and the damage degree of the two roadway ribs is quite different. Therefore, it is necessary to adopt an asymmetric partition gradually weakening support scheme for the roadway under the RCP and thoroughly follow the principle of solid support in critical areas and weak support in common areas to preserve the stabilization of the roadway surrounding rock.

### Anchor beam truss support principle

**Figure. 16:**
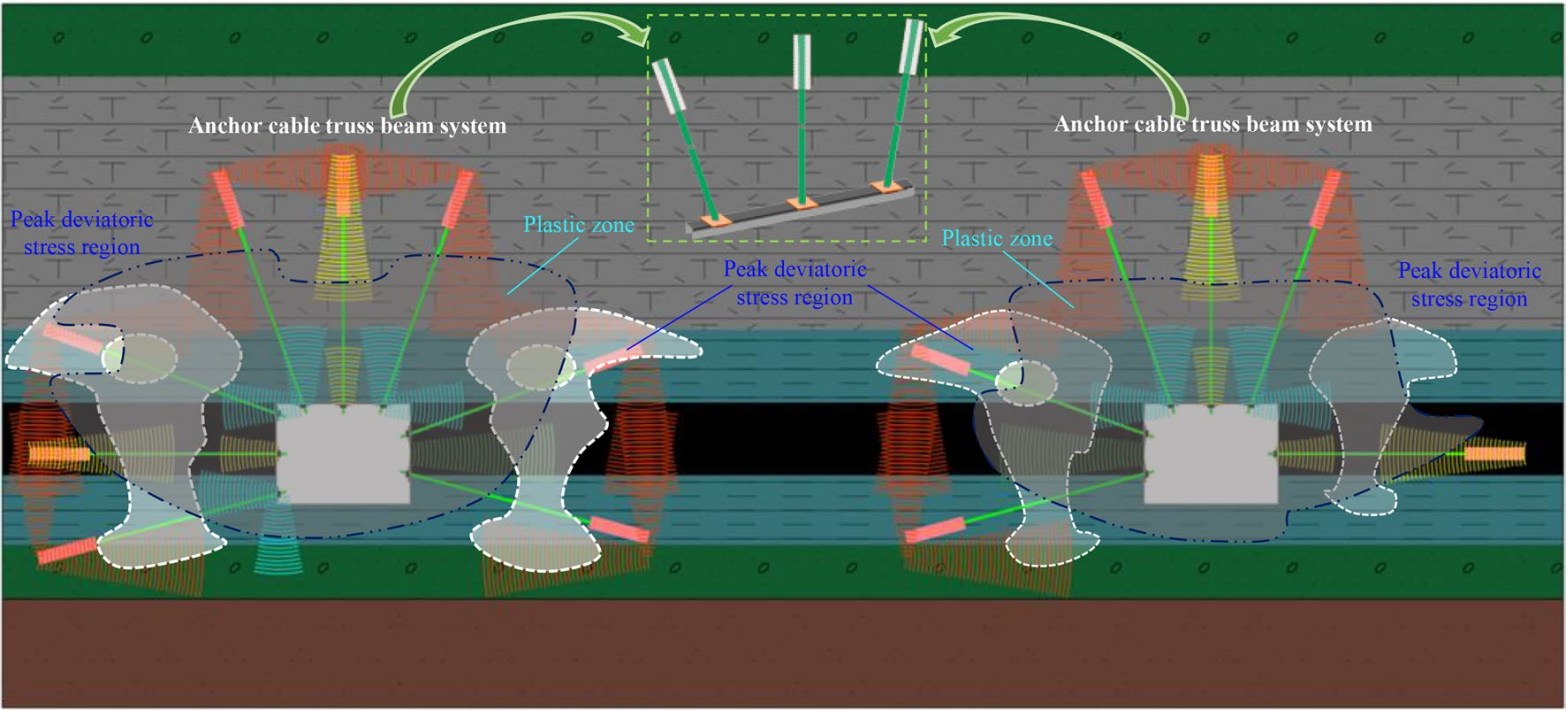
Control principle.

As illustrated in Fig. 16, Based on the damage situation of the surrounding rock in different partitions, the control core should focus on the peak region of deviatoric stress (when facing the next disturbance, it is more likely to be damaged). Use strong anchor cables to pass through the peak stress region, improve the stress state of the surrounding rock, and anchor it to stable/relatively stable rock layers. The overall support principle is as follows:

The anchor cable provides the deep anchorage zone.

The channel steel truss, the tray, and the anchor net provide shallow surrounding rock surface protection and pre-tightening force.

The channel steel connection is used to build a truss structure between the anchor cables, which can transmit the pre-tightening force in a combined form to the depth of the surrounding rock.

Located in the most seriously affected area within the scope of 20 m under the RCP, the support scheme needs to add high-resistance portal supports to resist large-scale roof subsidence, in addition to supporting components such as strong anchor cables.

Anchor beam truss system plays a key role in the roadway support system. The roof anchor beam truss system comprises channel steel joists and several high-strength anchor cables connected to it. The anchor cables at the two corners of the roof obliquely intersect at a certain angle, and the anchor cables in the roadway are vertically arranged. The oblique anchor cable can apply high pre-tightening force both horizontally and vertically. The horizontal component force can increase the tensile force of the surrounding rock at the shallow corner of the roadway roof and expand the extent of the deep anchorage area of the rock mass. The vertical component force and the vertical force imposed by the anchor cable work together to make the surrounding rock in a three-way compression state, forming a deep rock mass secondary bearing arch structure, fully utilizing the self-bearing capacity of the surrounding rock and improving the stability of the roof surrounding rock.

The horizontal component force and vertical component force are applied to the deep part of the surrounding rock by the anchor cable beam truss system of the mining side. The vertical component force makes the deep rock mass contact more closely. The inclined arrangement of the anchor cable at the side angle can enhance the capacity of the side to resist sliding shear. The horizontal component forces work together and spread to the deep anchorage zone, resulting in an effective compressive stress-bearing wall structure.

The double compressive stress-bearing wall structure is generated on two sides of the coal pillar under the combined action of the compressed area of the deep anchorage point of the coal pillar and the preload diffusion zone of the steel truss of the roadway surface. In the combined effect of bilateral compressive stress, the coal pillar changes from two-way compression to three-way compression, and the coal pillar's bearing capacity is improved, effectively preventing the coal pillar from crushing to the roadways on two sides under the high-stress state of dynamic-static superposition.

The overlapping influence area under the RCP is the most impacted by the stress concentration perturbation. The combined support scheme is adopted. The anchor cable beam truss system can stably anchor the shallow loose rock mass in the deep rock mass, and the three-way compression allows the self-bearing capacity of the surrounding rock to be utilized. However, the highly broken rock mass on the roadway surface has wholly lost the post-peak bearing capacity. The surface pre-tightening force offered by the anchor cable and other supporting components is finite, which is unable to form a self-bearing structure and is easy to lose and collapse. As a result, there is a need to add portal support to enhance the roadway surface's active support force. The advantage of portal support is that it can provide a high active support force, wide support range, and fit the roof of the roadway. The large deformation of the roof in area II under the RCP is serious, and adding portal support can effectively prevent the large settlement of loose rock mass of the roof.

### Asymmetric support scheme

**Figure. 17:**
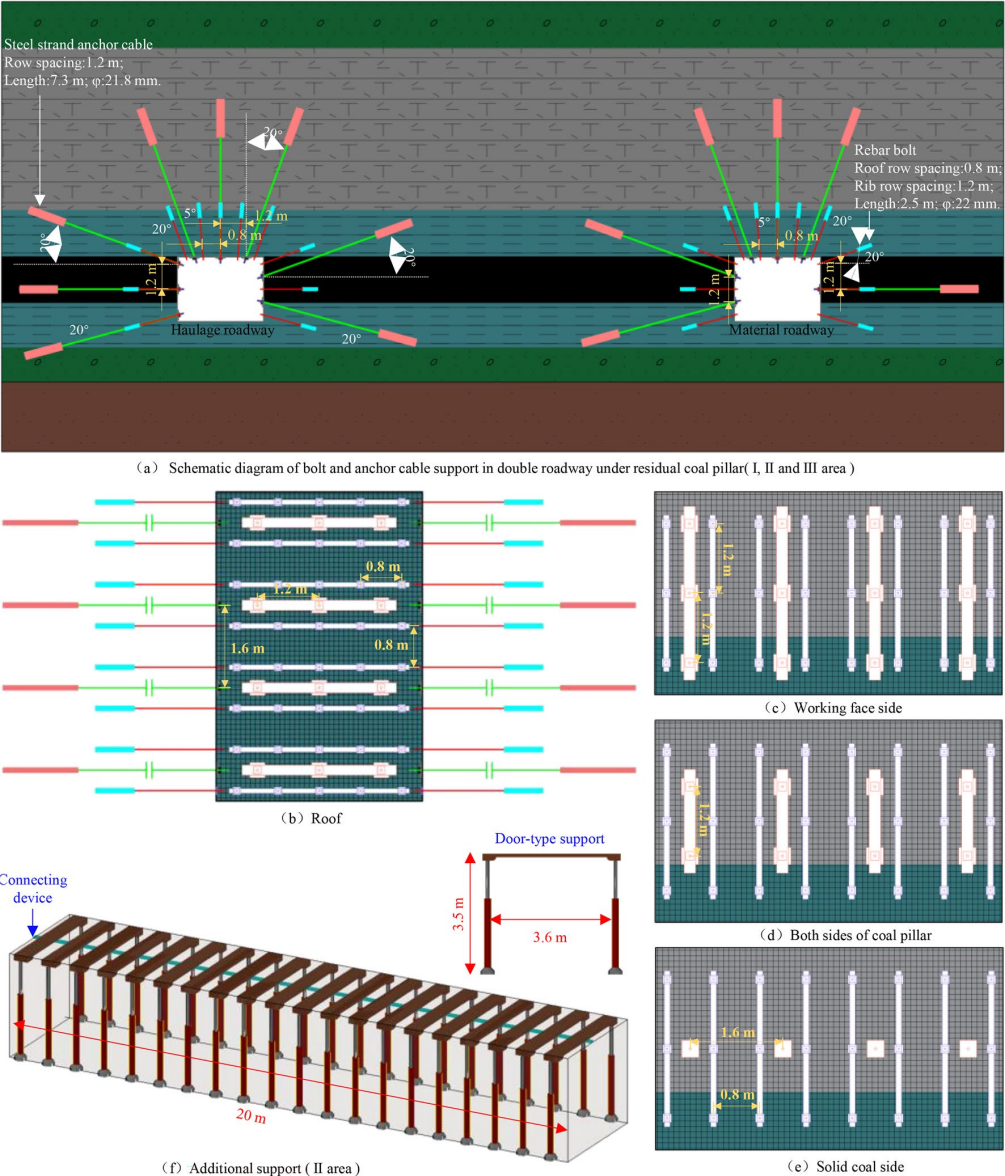
Design parameters of support.

The design parameters of double roadway excavation support under the RCP are illustrated in Fig. 17, and the selection of support material parameters is as follows:

① *Left-handed thread steel bolt* diameter of 22 mm, length of 2.5 m.
② *Steel strand anchor cable* diameter of 21.8 mm, length of 7.3 m.
③ *Portal support* ZT2 × 4000/23/50, support height 2.3–5.0 m, adaptation width 3.6 m.
④ *Steel ladder beam* 3400 mm × 100 mm × φ10 mm.
⑤ *Channel steel* The section size is 200 mm × 73 mm × 7.0 mm, and the length is 2800 mm.

The layout parameters of the bolts in the transportation and material roadways are the same. Three bolts are installed on the side, and five bolts on the roof, with a 150 mm × 150 mm square tray. The full-section bolt presses the φ10 mm steel ladder beam. It superimposes the φ4 mm steel welded mesh so that the single-point bolt support becomes the full-section bolt-mesh-beam combined support, giving full play to the support efficiency of the support components.

Three anchor cables are installed at the summit of the double roadway, three anchor cables are installed on the mining rib of the roadway, two anchor cables are installed on the coal pillar, and one anchor cable is arranged on the solid coal rib. Based on the influence degree of stress disturbance, it decreases from near to far. When the top and side are arranged with multiple anchor cables, a 150 mm × 150 mm square small tray, and a 300 mm × 300 mm square large tray are equipped. The small square tray presses 20# a channel steel truss and superimposes the sizeable square tray. The roof and side form an anchor beam truss system, which combines with the anchor beam support to form a unified support structure.

The range of area II (the overlapping influence area under the RCP) is 20 m, and the portal support is added in the interval, which supports elevation can be dynamically regulated. The distance between the supports is 1 m, and the working resistance can reach 40 MPa.

### supporting stress field

According to the above design support scheme and parameters, FLAC3D software simulates and studies the prestress distribution of anchor beam truss system + anchor net combined support.

**Figure. 18:**
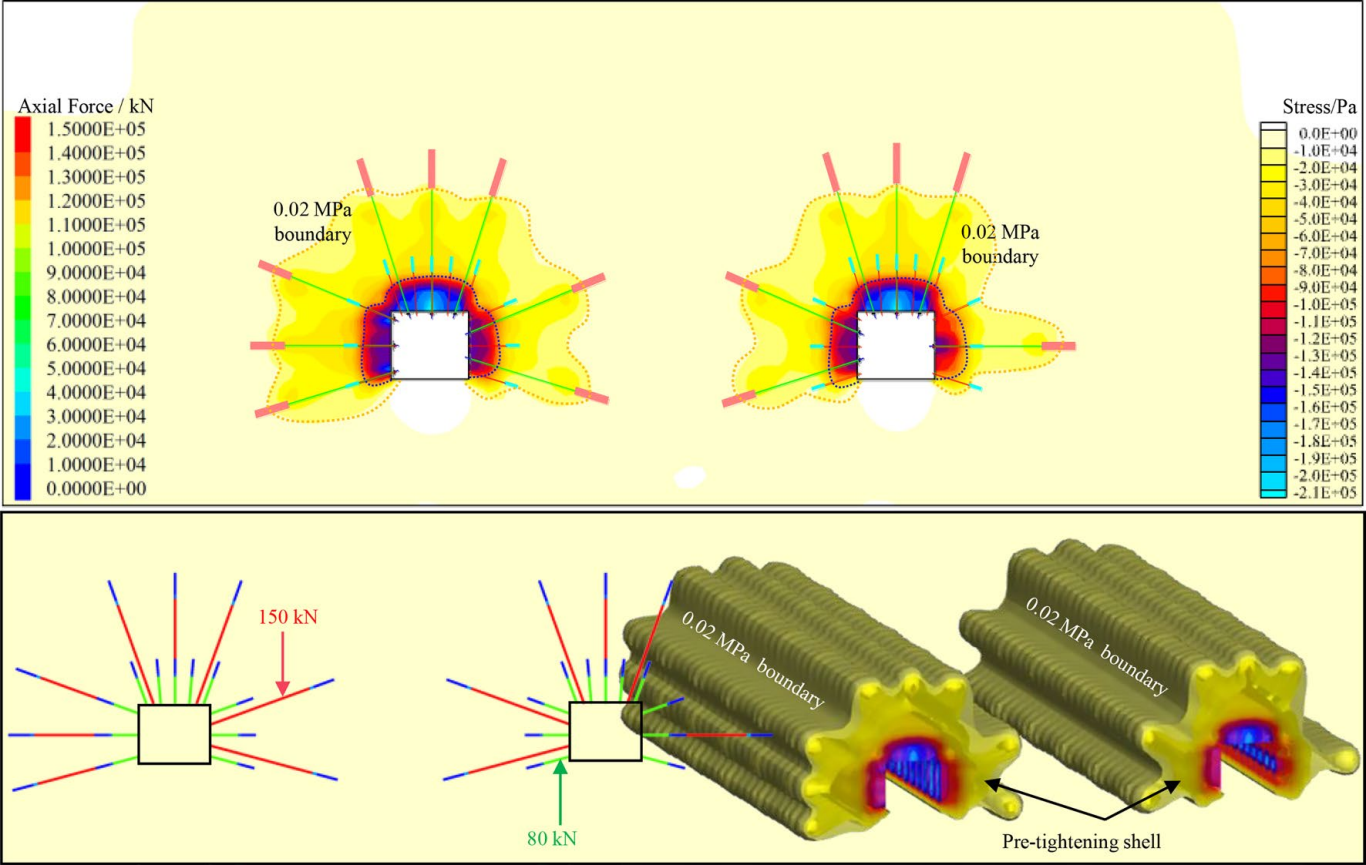
Schematic diagram of prestress distribution.

As illustrated in Fig. 18, in the prestress simulation study, 80 kN and 150 kN pre-tightening forces are applied to the anchor cable and anchor rod, respectively. In the combined effect of bolt and cable in a truss support system, the action of the force is transmitted between rock masses, and the prestress diffuses outward, which affects the stress of deep rock mass. Finally, a 0.02 MPa effective compressive stress outer boundary is formed in the anchorage zone towards the terminal end of the anchor cable, and a main bearing arch structure with 0.1 MPa high-pressure stress as the inner boundary is formed in the superficial surrounding rock. The prestressed anchor rod and the prestressed anchor cable are arranged jointly, and the two are coupled, and the deep rock mass is gradually loaded from the deep rock mass to the shallow rock mass. According to the strength of the compressive stress zone formed, the main bearing structure of the inner boundary and the second bearing structure of the outer boundary is formed step by step from the roadway’s surface to the deep part. The support strength of the whole support system is gradually changed, and the integrity and coupling are highly unified, which significantly improves the surrounding rock’s completeness and realizes the support system's optimization.

## Evaluation of on-site monitoring effect

### Working resistance of anchor cable (bolts)

**Figure. 19:**
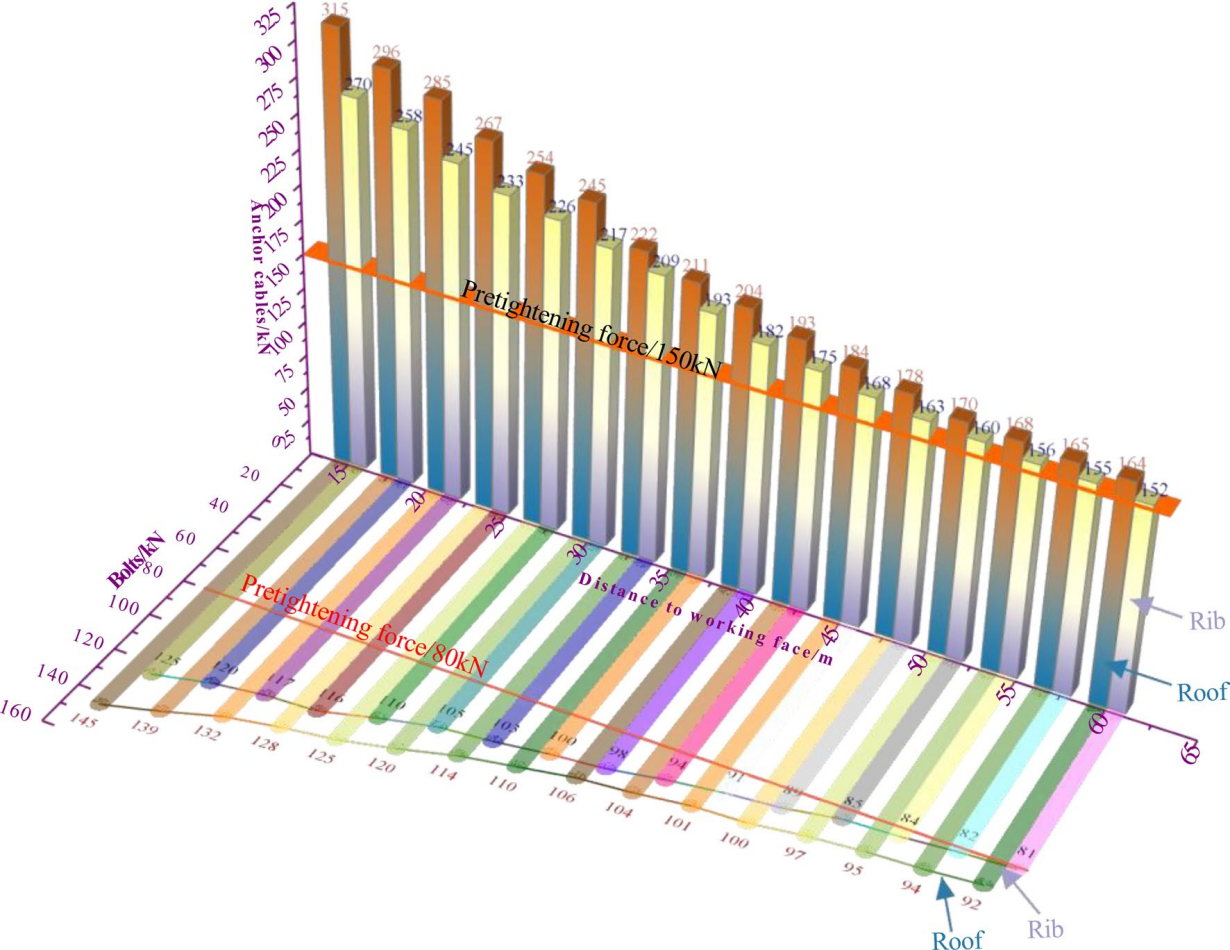
Stress of anchor cable (bolts) at different distances from panel.

As illustrated in Fig. 19, following the implementation of an unsymmetric zoning support scheme, key dangerous areas in haulage roadway are monitored—Variation trend of the distance between anchor cables (bolts) support resistance and panel in II area. Characterization of the working resistance curve of anchor cables (bolts):

1. When the distance from the panel is 45–60 m, the supporting resistance of anchor cables is higher than the initial pre-tightening force, but the variation is slight. The roof anchor cables went from 164 to 184 kN, the roof bolts went from 92 to 101 kN, the rib anchor cables went from 152 to 168 kN, the rib bolts went from 81 to 91 kN, and the changing trend was gentle.
2. However, when the separation to the panel is within 45 m, the distance from the panel decreases, and the support resistance of anchor cables increases significantly. The growth rate of the top anchor cable is greater than that of the rib anchor cable.
3. When 15 m from the panel, the working resistance of roof anchor cables reaches 315 kN, 270 kN of rib anchor cables, 145 kN of roof bolts, and 125 kN of rib bolts. The working resistance of roof anchor cable (bolts) is always higher than that of rib anchor cable (bolts).

The working resistance of anchor cables (bolts) is in a safe range, and the anchor cable beam truss system in II area can give sufficient play to its supporting property and fully enhance the rock bearing capacity. Even if influenced by advanced mining in the panel, the supporting components can still allow full play to safeguard the security and stability of the roadway.

### Deformation of surrounding rock

**Figure. 20:**
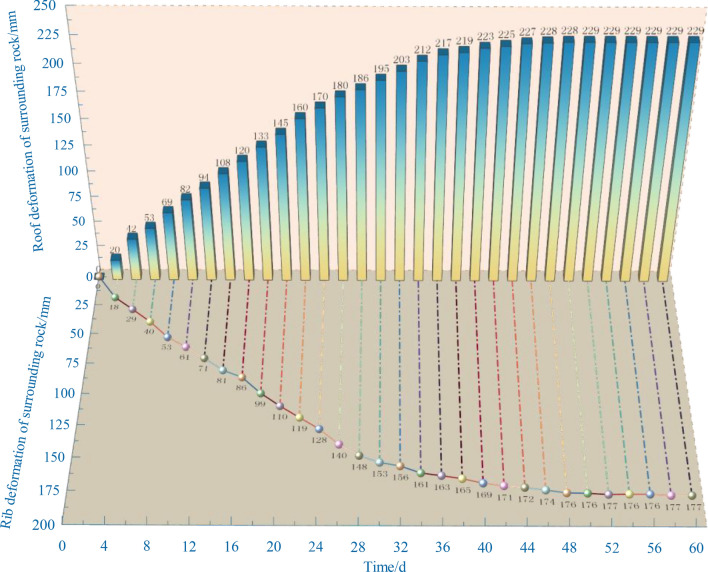
Distortion-time curves of surrounding rock.

As illustrated in Fig. 20, following the implementation of an unsymmetric zoning support scheme, the deformation of surrounding rock in a key dangerous area—Zone II of haulage roadway is observed for 60 days:

1. Based on trends in the surveillance curves, the distortion of the roadway roof and ribs is mainly formed in the first 35 days after the support is implemented. The distortion of the roof increases by 212 mm, and the distortion of the side increases by 161 mm, and then the increase of deformation tends to be flat.
2. In the last 25 days, the roof's deformation only augments 17 mm, and the side wall's distortion only augments 16 mm. Therefore, the deformation of the roof and the two ribs was well controlled by using key support methods in dangerous areas. The stabilization peak of the roof is 229 mm, compared to 177 mm for the ribs.

After the support reinforcement, the surrounding rock distortion is in a security domain and meets long-term and stable roadway use requirements. The observed results indicate that the support scheme can effectively control the large distortion of surrounding rock under the RCP, preserve the roadway stability, and satisfy the production use of the panel.

### Overall effect evaluation

Following implementing the unsymmetrical zoning sustain program, monitoring surrounding rock distortion brings out the average growth rate of roof deformation, which is about 6.1 mm/d, and the average growth rate of rib deformation is 4.6 mm/d within 35 days after the scheme's implementation. In the last 25 days, the average growth rate of both decreased to about 0.7 mm/d, and the deformation was finally in a stable state.

The distortion of the roof is greater than that of the ribs, but both of them are less than 230 mm, so the distortion of the surrounding rock is minor, and the roadway is secure and steady. According to the stress monitoring of the support system, the support resist of anchor cables and bolts began to gradually increase when it was 45 m distance to the panel. The roof anchor cable and bolt are finally stable at 315 kN and 145 kN, respectively, and the rib anchor cable and bolt are finally stable at 270 kN and 125 kN, respectively.

In addition, according to on-site feedback, after the disturbance of mining dynamic pressure came, the portal support did not appear to overpressure the unloading phenomenon, effectively reducing the roof deformation.

## Conclusion

Aiming at the problem of strong ground pressure in the roadway at the “+” intersection under the RCP in the CDCS, this paper introduces the deviatoric stress tensor index to analyze the failure characteristics and stress evolution law of surrounding rock. Based on the analysis results, the technical scheme of asymmetric and gradual weakening combined support in different regions is put forward to control the surrounding rock deformation of the roadway at the “+” intersection under the RCP. The conclusions are as follows:

1. The deviatoric stress of the RCP floor spreads symmetrically and decays with it. Its peak value appears on the floor of adjacent goaf, reaching 9.0 MPa. The deviatoric stress in the underlying range of 9 m is double-humped, and the peak value decays from 8.6 to 7.4 MPa, then changes to a single-humped shape.
2. After excavating the roadway, the range of plastic failure and the degree of deviatoric stress concentration in the mining roadway peak at a distance of − 10 m from the RCP. The maximum range of the plastic zone of the roof and two ribs is 3.4 m and 5 m, respectively, and the peak value of deviatoric stress is 10 MPa. The peak zone of deviatoric stress evolves from the roof to the two ribs and the sharp corner with the distance from the RCP decreasing (10 m → 0 m → − 10 m). The shape presents the evolution law from the “ellipse” of the top plate → the “crescent” of two ribs → the “cochlea” of the tips of the ribs.
3. When the distance from the RCP is 10 m, 0 m, − 10 m, − 20 m, and − 30 m, the peak value of deviatoric stress in two ribs of the double roadway shows a decreasing trend along the panel side → section coal pillar side → solid coal side. Based on this, the underlying 45 m of the RCP is divided into area I (10 m), area II (overlapping area 20 m), and area III (15 m) according to the influence degree of superimposed stress.
4. The technical scheme of asymmetric and gradual weakening combined control of roof-rib channel steel truss anchor cable + door-type support in area II is implemented. The peak value of roof deformation is 229 mm, and rib deformation is 177 mm. The working resistance of the anchor cable (bolts) is normal. It successfully resisted the large deformation of the roadway caused by superimposed stress disturbance under the RCP.

## Data Availability

All data generated or analysed during this study are included in this published article.
